# Widespread platinum anomaly documented at the Younger Dryas onset in North American sedimentary sequences

**DOI:** 10.1038/srep44031

**Published:** 2017-03-09

**Authors:** Christopher R. Moore, Allen West, Malcolm A. LeCompte, Mark J. Brooks, I. Randolph Daniel, Albert C. Goodyear, Terry A. Ferguson, Andrew H. Ivester, James K. Feathers, James P. Kennett, Kenneth B. Tankersley, A. Victor Adedeji, Ted E. Bunch

**Affiliations:** 1 Savannah River Archaeological Research Program, South Carolina Institute of Archaeology and Anthropology, University of South Carolina, P.O. Box 400, New Ellenton, SC 29809, USA; 2 GeoScience Consulting, Dewey, AZ, 86327, USA; 3 Center of Excellence in Remote Sensing Education and Research, Elizabeth City State University, Elizabeth City, NC 27921, USA; 4 Department of Anthropology, East Carolina University, Greenville, NC 27858, USA; 5 South Carolina Institute of Archaeology and Anthropology, Columbia, SC 29208, USA; 6 Department of Environmental Studies, Wofford College, 429 N Church Street, Spartanburg, SC 29303-3663, USA; 7 Department of Geosciences, University of West Georgia, 1601 Maple Street, Carrollton, GA 30118, USA; 8 University of Washington, Luminescence Dating Laboratory, 125 Raitt Hall, Seattle, WA 98195-3412, USA; 9 Department of Earth Sciences and Marine Science Institute, University of California, Santa Barbara, USA; 10 Departments of Anthropology and Geology, University of Cincinnati, Cincinnati, OH 45221, USA; 11 Department of Natural Sciences, Elizabeth City State University, Elizabeth City, NC 27909, USA.; 12 Geology Program, School of Earth Science and Environmental Sustainability, Northern Arizona University, Flagstaff, AZ 86011, USA.

## Abstract

Previously, a large platinum (Pt) anomaly was reported in the Greenland ice sheet at the Younger Dryas boundary (YDB) (12,800 Cal B.P.). In order to evaluate its geographic extent, fire-assay and inductively coupled plasma mass spectrometry (FA and ICP-MS) elemental analyses were performed on 11 widely separated archaeological bulk sedimentary sequences. We document discovery of a distinct Pt anomaly spread widely across North America and dating to the Younger Dryas (YD) onset. The apparent synchroneity of this widespread YDB Pt anomaly is consistent with Greenland Ice Sheet Project 2 (GISP2) data that indicated atmospheric input of platinum-rich dust. We expect the Pt anomaly to serve as a widely-distributed time marker horizon (datum) for identification and correlation of the onset of the YD climatic episode at 12,800 Cal B.P. This Pt datum will facilitate the dating and correlating of archaeological, paleontological, and paleoenvironmental data between sequences, especially those with limited age control.

In 2013, Petaev *et al*.^1^ discovered an anomalously large platinum (Pt) peak in ice core samples from the Greenland Ice Sheet Project 2 (GISP2), thus providing the most compelling evidence to date for a catastrophic extraterrestrial event coincident with the onset of the Younger Dryas (YD) climate episode. In the study by Petaev *et al*., high-resolution (2.5–4.6 y) time-series of ice core samples were analyzed for trace and major element concentrations using inductively coupled plasma mass spectrometry (ICP-MS). Petaev *et al*.^1^ reported the presence of a Pt peak anomaly at the Bølling-Allerød/Younger Dryas Boundary (YDB), coincident with a large shift in δ^18^O values, confirming the onset of cooler conditions at the beginning of the YD interval. This peak interval is represented by a rise in Pt concentrations over 14 years and subsequent drop during the following 7 years, consistent with the known residence time of stratospheric dust^1^. This sharply defined Pt anomaly at the YD onset in GISP2 is coeval with other YDB impact-related proxies, including nanodiamonds and melted spherules, found in Greenland and across four continents and is proposed by Petaev *et al*. to have resulted from a highly fractionated, Ir-deficient, iron-rich, extraterrestrial impactor. However, ten additional YDB studies have reported different concentrations and ratios of Pt and other platinum group elements (PGE), including iridium (Ir), osmium (Os), ruthenium (Ru), and rhodium (Rh), all of which usually co-vary (Supplementary Information, “Summary of PGE Occurrence in the YDB”). These other studies do not rule out an extraterrestrial impactor as source of the PGE anomalies, but do not support the conclusion that it was highly fractionated and Ir-deficient, leaving open the question of the exact nature of the Pt source (see Supplementary Information, “Potential Sources of YDB Platinum”).

Sawlowicz^2^ notes that PGE anomalies may result from multiple processes of enrichment, including: a) cometary or meteoritic influx [Supplementary Table 7]; b) impactites from an extraterrestrial impact event [Supplementary Table 7]; c) volcanoes [Supplementary Table 10]; d) mantle material, e.g., from tectonic motion or in cratons [Supplementary Table 7]; e) exhalative-hydrothermal processes; f) precipitation from seawater; g) post-depositional transport and precipitation at redox boundaries; and h) reduction from intermediate and low-temperature solutions. In this study, we evaluate evidence of PGE enrichment from archaeologically-stratified sedimentary sequences across North America that date to the YDB in order to test the implications of Petaev *et al*.^1^ who suggested the likelihood of a global Pt anomaly. Results are discussed below and in Supplementary Information.

The Younger Dryas impact hypothesis proposed a causal link between a cosmic impact event and a) the onset of the YD climate cooling episode at ~12,800 calendar years BP, b) a peak in continental-scale biomass burning, c) extinction of more than 35 genera of North American Pleistocene megafauna, and d) the demise of the Paleoindian Clovis technocomplex^3^. In support of those links, several studies^3^,^4^,^5^,^6^,^7^ have reported an exotic assemblage of impact-related proxies in a widely-distributed layer at the YDB, dating to 12,800 ± 150 Cal B.P. Impact proxies reported for YDB sites include but are not limited to high-temperature iron and silica-rich magnetic spherules, nanodiamonds, aciniform carbon (soot), high-temperature melt-glass, and elevated, above-background concentrations of nickel, osmium, and iridium^3^,^4^,^5^,^6^,^7^. Researchers have also hypothesized a human population decline or demographic shift immediately following the disappearance of the Clovis technocomplex at the YD onset^8^,^9^,^10^,^11^.

A number of other studies have made critical assessments of the evidence for a YD impact event. Specifically, questions have been raised regarding the nature or replicability of the reported impact proxies, the accuracy of the established chronology, and its purported effects on humans and animals at the YD onset^12^,^13^,^14^,^15^,^16^,^17^,^18^. All of these criticisms have been addressed by additional and independent studies replicating the original findings of Firestone *et al*.^3^,^7^,^19^,^20^. Another investigation demonstrated the synchroneity of the YDB layer throughout its geographic range based on Bayesian modeling of data from 23 stratigraphic sections and 354 dates from 12 countries^21^. The modeled YDB age range is 12,835–12,735 Cal B.P. (at 95% probability). In one study critical of the YDB hypothesis, Paquay *et al*.^22^ reported small YDB PGE peaks in Pt, Ir, and Os at Murray Springs, AZ and Lake Hind, AB, Canada, co-occurring with peaks in YDB microspherules and nanodiamonds. That study, claiming the PGE anomalies merely resulted from natural authigenic enrichment, dismisses an extraterrestrial origin, but without citing evidence. Instead, even though their Pt anomalies are small, their results are consistent with the independent impact-related Pt study by Petaev *et al*.^1^.

Of particular relevance to our study, Andronikov *et al*.^23^,^24^,^25^ investigated sediments from Belgium, the Netherlands, Lithuania, and NW Russia near Finland, reporting sharp YDB enrichment in Pt at the YD onset, as well as other meteoritic elements such as nickel, chromium, copper, and iridium. In a separate study, Andronikov *et al*.^19^ analyzed YDB magnetic microspherules from Blackwater Draw, New Mexico using scanning electron microscopy (SEM), electron probe microanalysis (EPMA), X-ray diffraction (XRD), and laser-ablation inductively coupled-plasma mass spectrometry (LA-ICP-MS). They reported an abundance peak in YDB microspherules that display melted, dendritic textures, confirmed through a combination of SEM and energy dispersive spectroscopy (EDS). These results confirm those for YDB spherules from Blackwater Draw by Firestone *et al*.^3^ and LeCompte *et al*.^20^, and contradict the spherule results of Surovell *et al*.^18^, who found no peak, but neglected to perform the SEM analyses required to correctly identify YDB spherules.

In the same study of Blackwater Draw, Andronikov *et al*.^19^ reported that four of six YDB spherules contained very high Pt abundances, ranging from 18.2 to 460 ppb, up to 900× crustal abundance of 0.5 ppb^26^, consistent with the finding of elevated YDB Pt in the Greenland ice core by Petaev *et al*.^1^ (Supplemental Table 8). For two spherules, Pt was below detection. In addition, five of the six Blackwater Draw YDB spherules contained elevated concentrations of Ir at >230× crustal abundance of 0.022 ppb^26^, supporting the findings of Firestone *et al*.^3^. They also found high concentrations of YDB Os at >240× crustal abundance of 0.031^26^, supporting the findings of Wu *et al*.^27^. The presence of PGE-enriched spherules heterogeneously distributed throughout any given YDB sample could account for the highly variable PGE concentrations reported by Firestone *et al*.^3^, who proposed that variability to result from a PGE-rich “nugget effect”.

Andronikov *et al*.^19^ reported that Pt, Ir, and Os values were near detection limits for spherules, and therefore, have high uncertainties. For comparison, they analyzed six YDB magnetite and ilmenite grains, which showed undetectable Ir in grains compared to an average of 2.2 ppb in YDB spherules, undetectable Os versus 4 ppb, and an average of 16.2 ppb of Pt in non-YDB grains compared to 145 ppb in the YDB. Such large compositional differences between grains and spherules suggest that PGE enrichments in the YDB spherules are real and that the spherules did not derive from the melting of local magnetic and/or ilmenite grains, but rather are of non-local origin. Even though Pt has been shown to be present in YDB spherules, they may not be the only Pt source. There is currently no evidence to preclude the possibility that high Pt concentrations may also be present in non-spherulitic YDB grains and dust.

## Study Sites

Following the discovery of the Pt anomaly at the onset of the YDB in the Greenland ice sheet^1^, it was hypothesized that it should exist in terrestrial sedimentary sections of the same age across North America. Key objectives of this study are a) establishing whether or not a Pt anomaly exists in terrestrial sediments of YD age that is similar to that reported from the GISP2 ice core, and b) if found, determining if it can be used to identify the YDB in other sites lacking precise age control. It was beyond the scope of this study to identify the carrier and source of the Pt, if present.

**Figure 1:**
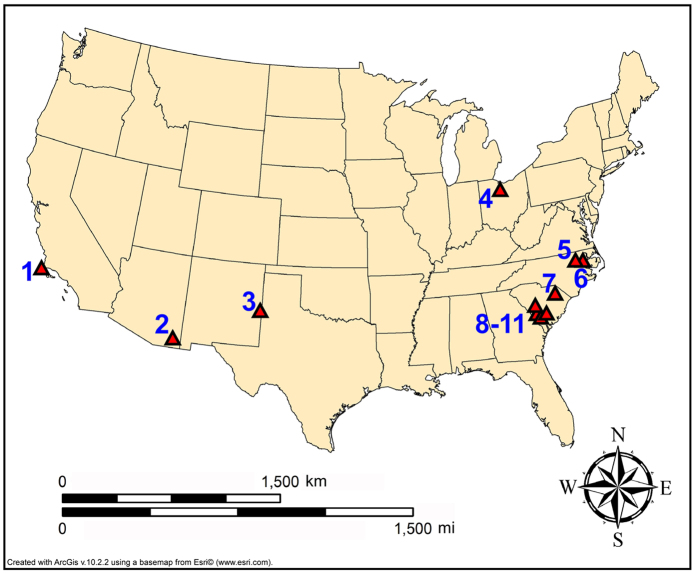
Map showing study sites tested for platinum (Pt) and palladium (Pd): (#1) Arlington Canyon, Santa Rosa Island, California; (#2) Murray Springs, Arizona; (#3) Blackwater Draw, New Mexico; (#4) Sheriden Cave, Ohio; (#5) Squires Ridge, North Carolina; (#6) Barber Creek, North Carolina; (#7) Kolb, South Carolina; (#8) Flamingo Bay, South Carolina; (#9) Pen Point, South Carolina; (#10) Topper, South Carolina; (#11) Johns Bay, South Carolina. Map generated with ArcGIS software (v.10.2.2) using an Esri© basemap of the United States.

We first tested the hypothesis of a widespread YDB peak in Pt from well-dated, well-stratified sites with clearly established YD-age sediments and with previously reported YDB impact proxies (e.g. microspherules and nanodiamonds). These sites include Arlington Canyon on Santa Rosa Island in Southern California, Murray Springs in southeastern Arizona, Blackwater Draw in eastern New Mexico, and Sheriden Cave in Ohio (Fig. 1). We hypothesized that if Pt anomalies are present in these well-dated sites of YD age, then the Pt anomaly may serve as a widely-distributed time marker or datum for the YD onset. After testing our initial well-dated sites for Pt, we investigated other less well-dated and undated sites that have good chronostratigraphic and/or archaeostratigraphic control. These sites include Squires Ridge and Barber Creek in eastern North Carolina, Kolb in northeastern South Carolina, and Flamingo Bay, Pen Point, Topper, and Johns Bay in southeastern South Carolina (see Fig. 1 and Supplementary Information, “Study Sites”).

Lithologies for western and Midwestern study sites have been well-described elsewhere, but vary widely from cobble lag and alluvial sands and gravels at Arlington Canyon, incised marl deposits filled with stream-channel sands and gravels at Murray Springs, Pleistocene sandy alluvium capped by diatomite and silty muds at Blackwater Draw, and deeply buried, stratified deposits within a collapsed karst cavern at Sheriden Cave (see Supplementary Information, “Study Sites”). Organic rich black mats are only present at western and Midwestern study sites and have been addressed elsewhere^28^. Black mats have previously been claimed to be an indirect impact marker, related to environmental degradation resulting from impact-related abrupt YD climate change, but that issue is beyond the scope of this paper. Lithologies for eastern sites consist uniformly of undifferentiated medium to fine sand from diverse depositional environments in the South Atlantic Coastal Plain. These include linear sand ridges of mixed aeolian and fluvial origin, aeolian sand-sheets and dunes, levee and alluvial terrace deposits, slopewash deposits, and lacustrine and aeolian Carolina bay sand rims. None of the eastern sites are associated with the black mat or any measurable increase in organic matter (see Supplementary Information, “Study Sites”).

## Results and Discussion

**Figure 2:**
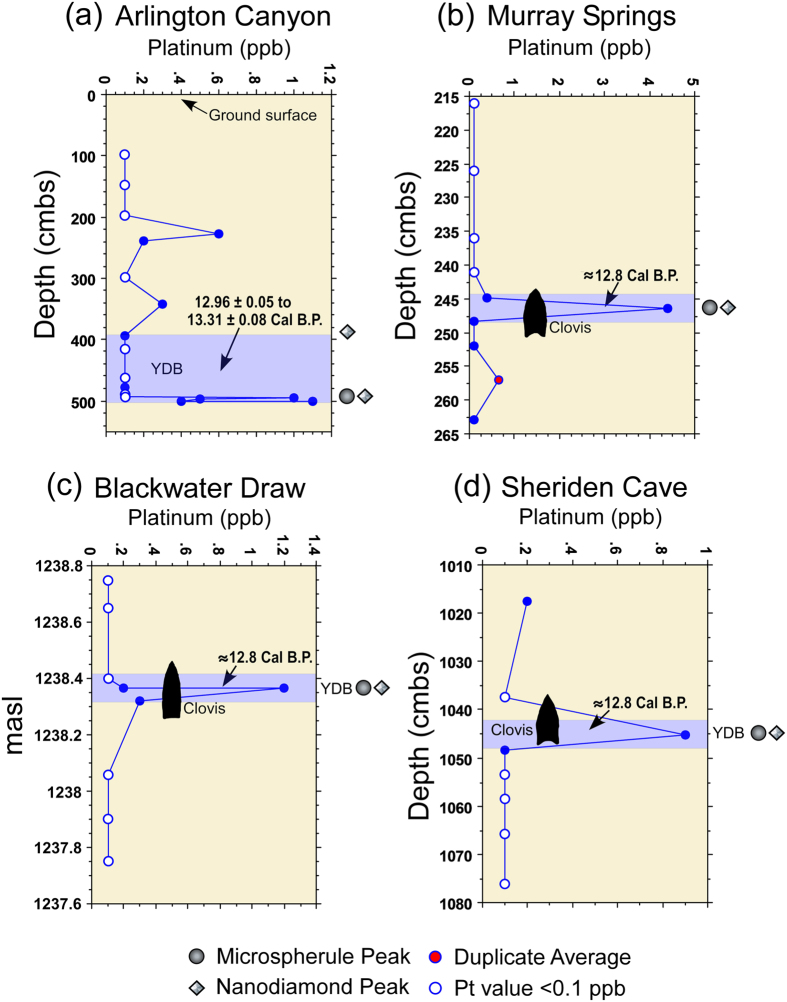
Site graphs for western and Midwestern study sites (**a**–**d**). Graphs show abundance in ppb (error = +/−0.1 ppb), archaeostratigraphic data (Paleoindian Clovis hafted biface silhouettes), radiocarbon dates (calibrated B.P.), microspherule and nanodiamond peaks^3^,^5^,^6^,^7^, and interpreted YDB. Each sample is plotted in the middle of the sample interval. The chronostratigraphic position of the YDB for each site was determined based on (**a**) linear interpolation of 12 AMS dates; (**b**) interpolation of 7 conventional and AMS radiocarbon dates based on second-order polynomial regression; (**c**) logarithmic interpolation of 5 conventional and AMS radiocarbon dates and the stratigraphic position of temporally diagnostic hafted bifaces; and (**d**) 3 AMS dates selected from the YDB layer^7^. A Bayesian analysis of dates from all western and Midwestern study sites demonstrates synchronous deposition of the YDB layer within the limits of dating uncertainty (~100 y)^21^. See Supplementary Figures 4–7 and Supplementary Table 3 for more detail on stratigraphy and dating for sites.

**Figure 3:**
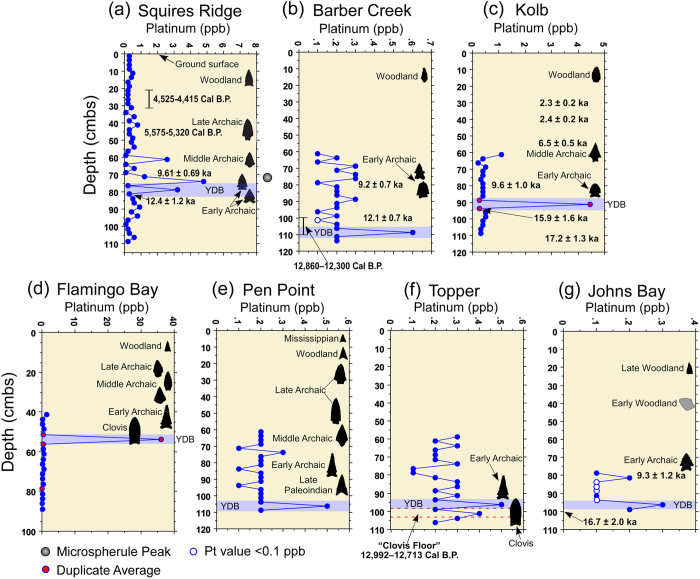
Site graphs for eastern study sites (**a**–**g**). Graphs show platinum (Pt) abundance in ppb (error = +/−0.1 ppb), generalized archaeostratigraphic data (Paleoindian through Woodland hafted biface silhouettes), chronometric dates (OSL [ka] and radiocarbon [Cal B.P.]), depth of microspherule peak (Squires Ridge), and interpreted YDB. Each sample is plotted in the middle of the sample interval. (**a**) Radiocarbon date from Level 11 (100-110 cmbs) at Barber Creek is from an adjacent excavation block. (**b**) Radiocarbon date from Clovis occupation surface “Clovis Floor” at Topper is from an adjacent excavation block. In a paper by Kennett *et al*.^21^, a Bayesian analysis of dates from Topper and Barber Creek demonstrated synchronous deposition of the YDB layer within the limits of dating uncertainty (~100 y)^21^. See Supplementary Figures 9–15 and Supplementary Tables 4–6 for more detail on stratigraphy and dating for sites.

Initially, it was not clear that the anomaly would be conspicuous in terrestrial sedimentary sequences, because the chrono-stratigraphic resolution available to us was lower than that available during the GISP2 investigation^1^. Testing was limited at first to four North American sedimentary sequences (Arlington Canyon, California; Murray Springs, Arizona; Blackwater Draw, New Mexico; and Sheriden Cave, Ohio; Fig. 1) with well-defined and well-dated YDB age sediments containing peaks in YDB impact-related proxies. These analyses revealed a large above-background Pt anomaly at each site in the identical sample previously identified as the YD boundary layer (Fig. 2) containing abundance peaks in YDB proxies, including micro-spherules, meltglass, and nanodiamonds. This encouraged us to extend Pt analyses to seven other stratified archaeological sites in eastern North America (Fig. 3). Thus, sediment samples were collected from 11 sites with established sequences based on archaeostratigraphy and/or chronometric evidence that suggested the possible presence of the lower boundary of the YD Chronozone.

Results that are reported here for 11 sites from six U.S. states, from the Atlantic to Pacific coasts, provide strong evidence for above-background enrichment in Pt within sediments that date to the onset of YD climate change at ~12,800 Cal B.P. (Figs 2 and 3; Supplementary Tables 1 and 2). Pt abundances from our study sites averaged 6.0 ppb (range: 0.3 to 65.6 ppb) compared to background abundances above and below the YDB layer averaging 0.3 ppb. Average background Pt concentrations are all lower than crustal abundance of 0.5 ppb, whereas average YDB concentrations are 12× higher. These concentrations are also higher than the peak Pt concentration (~80 parts per trillion [ppt] or 0.1 ppb) reported at high chronological resolution from the GISP2 ice-core in Greenland by Petaev *et al*.^1^. All study sites contain significant Pt peaks that are ~3 to 66× higher than in Greenland, possibly because a) Greenland was further from proposed North American impact sites, b) atmospheric circulation from North America carried less Pt to Greenland, c) deposition and preservation of Pt and other impact proxies are highly variable, d) normal Pt concentrations found in sediment add a background level upon which the YDB Pt concentrations are superimposed, e) Greenland ice accumulated at a faster rate than terrestrial sediment, diluting the relative Pt concentrations, and/or f) the lower resolution of this study’s samples may be roughly equivalent to the total input of Pt to Greenland across ~21 years. This wide variation in Pt concentrations among sites is possibly due to post-depositional processes such as winnowing and erosion. In addition, discontinuous sampling at a few sites and the accidental crosscutting of invisible depositional boundaries during sampling means that the highest value of Pt may not have been obtained.

We also investigated the heterogeneous distribution of Pt (i.e., nugget effect) within each sample and found that Pt concentrations may vary widely between duplicate testing of the same aliquot (Supplementary Information “Heterogeneous Distribution of Pt and Pd”). For example, the maximum YDB Pt difference occurred at the Flamingo Bay site, where replicate analyses of the same sample measured concentrations of 6.4 and 65.6 ppb. Replicate values for samples both above and below the Pt anomaly at Flamingo Bay were uniformly low with Pt concentrations at 0.5 and 0.4 ppb, indicating a nugget effect only in the YDB sample at this site. For this reason, future studies, wishing to use the Pt anomaly as a potential YDB datum, should include the analysis of duplicate samples across the YDB in order to verify the presence or absence of the Pt anomaly reported here.

At 3 of the 4 western and Midwestern study sites, the combination of archaeostratigraphic data and radiocarbon dating constrain the timing of Pt anomalies only to the onset of the YD climate episode. At Arlington Canyon, a Pt anomaly occurs above the YDB; however, multiple anomalies and the largest anomalies are constrained to the YDB interval. More on this below. For less well-dated eastern sites, a combination of archaeostratigraphic data along with luminescence and radiocarbon dating provide evidence consistent with the placement of Pt anomalies at the YD onset at 6 of the 7 study sites. For the remaining eastern site, Squires Ridge, multiple abundance peaks in both Pt and high-temperature YDB magnetic spherules (Fig. 3a) begin at the YD onset and continue upward for ~25 cm into post-YD-age sediments. This observation is consistent with known reworking of sediment at this site by aeolian and fluvial processes after ca. 12,800 Cal B.P.^29^. The deepest and earliest Pt anomalies at Squires Ridge occur in sediment samples dated by optically stimulated luminescence (OSL) to overlap the YD age interval at 2-sigma (13,600–11,200 ka). Even though the Pt anomaly begins at the YD onset, as expected, and thus, is a useful time marker in the majority of cases, the blurring of the Pt record at this site underscores the complex taphonomic processes typical of such archaeological sites. Thus, given the known post-depositional processes common to these sites, it should be no surprise if some sedimentary sequences contain no Pt anomalies, while others contain multiple anomalies. Based on this study, it appears that the occurrence of either possibility is uncommon. Rather, the typical pattern exhibits a single Pt anomaly at the YDB, none above the YDB, and none within undisturbed sediments predating the YDB event.

Because of the presence of multiple Pt anomalies at Squires Ridge and Arlington Canyon, we increased the number of samples tested for other study sites, both above and (where available) below the inferred YDB. Although the record at two YDB sites is unclear, 9 out of 11 study sites have Pt anomalies that are constrained to or consistent with the YDB layer based on chronometric dating and/or archaeostratigraphy, thus, making the Pt anomaly a useful time marker horizon. A few other study sites have samples with elevated Pt in post-YDB sediments; however, with only one exception, they represent only slight deviations from background. Although Arlington Canyon has a relatively large Pt anomaly above the YDB, the sampling location is reported to be a catchment basin filled with reworked material, and therefore, would be expected to contain reworked YDB Pt. Multiple and significantly higher Pt anomalies are present within the established YDB interval at Arlington Canyon and are associated with other known impact proxies (see discussion of Arlington Canyon in Supplementary Information, “Study Sites”). Multiple Pt anomalies at Squires Ridge and Arlington Canyon suggest that the use of this anomaly as a time marker for the onset of the YD in the absence of precise dating or other YDB markers, must be evaluated carefully and with a clear understanding of the sedimentary and taphonomic processes operating at each site. Using Pt as a time marker is subject to the same limitations as radiocarbon and OSL dating, in which redeposition of materials (e.g., charcoal) upward or downward in the sedimentary column affects the accuracy of age-depth models. Consequently, Pt concentrations must be used cautiously as a time marker, and it is advisable to utilize additional dating methods as well.

We acknowledge that more refined chronological controls are needed for some eastern sites before the YDB/Pt anomaly correlation can be established with absolute certainty. Although absolute dating for tested sedimentary sequences at Flamingo Bay and Pen Point are lacking, archaeostratigraphic data provide relative chronological control and Pt anomalies are consistent with the location of the YDB onset based on absolute dating of virtually all other study sites. There is no evidence of fluvial reworking of archaeologically-stratified terrace deposits at Pen Point, and cultural sequences are found from Late Paleoindian through Mississippian occupations, indicating a high degree of site integrity. Multiple Clovis and other Paleoindian tools recovered from Flamingo Bay also provide useful chronostratigraphic anchors in the absence of precise chronometric dating. The presence of Early Archaic artifacts in the same levels as Clovis at Flamingo Bay indicates that these surfaces were stable to slowly accreting for several millennia before being buried incrementally through a combination of slopewash and aeolian accretion, as described in Supplementary Information, “Study Sites”.

For some eastern sites, reliance on archaeostratigraphic data as a relative stratigraphic marker (in the absence of absolute dating) is based on numerous studies by multiple authors of artifact distributions, the stratigraphic position of temporally diagnostic artifacts (i.e., artifacts with well-established chronologies), intact cultural features, occupation surfaces indicated by dense lithic debris, refitting studies indicating minimal vertical displacement, and analysis of sediments indicating discrete periods of sedimentation, artifact burial, and pedogenesis^29^,^30^,^31^,^32^,^33^,^34^,^35^,^36^. Limited bioturbation and sediment reworking is common and expected in sandy sites, but appears to be highly variable spatially, and for eastern study sites, represents an overprint of the relatively intact archaeostratigraphic character of the sites rather than as the principle mechanism for artifact burial^29^. While occasional stratigraphic inversions of temporally diagnostic artifacts and vertically displaced refits occur, the presence of discrete occupation floors or zones for these study sites is clear upon examination of piece-plot data for large excavation blocks or trenches^30^,^31^. Although use of archaeostratigraphic data for relative dating should be applied with caution, its use here is justified based on intensive geoarchaeological investigations and refitting studies and is consistent with chronologies developed from stratified sequences in similar environments where we do have absolute dates.

Another anomaly involves the Pt/Pd ratios for the YDB layer, which are typically very different from the background Pt/Pd ratios above and below the YDB layer (Supplementary Figs 3 and 8). Pt/Pd anomalies are present at 8 of the 11 study sites for the YDB layer. Smaller or secondary Pt/Pd anomalies are present at Flamingo Bay and Johns Bay in the YDB layer while Barber Creek is the only study site that lacks a Pt/Pd anomaly in the peak deposition layer. Because there is no known geochemical reason that Pt/Pd ratios should differ locally only in the YDB, Pt/Pd anomalies suggest the influx of non-local Pt-enriched material 12,800 years ago. The wide distribution of Pt-rich sites suggests the causal mechanism is some exogenic, continent-wide process, including the possibility of an extraterrestrial impact, as supported by the presence of impact proxies such as magnetic microspherules and nanodiamonds, which occur in the same samples as the Pt and Pt/Pd anomalies for study sites where data on these proxies are available^3^,^4^,^5^,^6^,^7^.

Regarding the likely mechanisms for PGE enrichment, discussed in Sawlowicz^2^, the age, location, elevation, and geomorphology of our study sites precludes precipitation from seawater and exhalative-hydrothermal processes as possibilities for Pt anomalies. The distribution of Pt from mantle material only into the YDB layer across North America and Eurasia is implausible through any normal terrestrial process. Similarly, post-depositional leaching, transport, and precipitation at a redox boundary do not seem to be at play because of the poor stratigraphic correlation (Supplementary Figure 16 and Supplementary Table 9) between Pt and the fines (silt and clay-sized particles). Volcanism is ruled out due to previous geochemical studies demonstrating a lack of tephra or sulfur anomalies^3^,^4^,^7^ associated with Pt in the YDB layer, and there is no evidence for continental-scale volcanism at the YD onset [Supplementary Table 10]^1^. In addition, there are no studies of authigenic enrichment that can account for the high concentrations of Pt found at many of our study sites, which include widely different depositional environments (Supplementary Tables 3 and 4) and rates of sedimentation. This leaves only atmospheric input through an extraterrestrial impact as the most plausible source of Pt enrichment (Supplementary Information, “Potential Sources of YDB Platinum”). However, more work is needed to confirm or exclude an extraterrestrial impact event origin and to determine if microspherules and/or Pt-rich dust are the carriers of Pt and other PGEs in the YDB layer.

The platinum anomalies reported here for eastern sites are particularly striking, given the difficulties in establishing archaeological stratigraphy and landform chronologies for the relatively uniform, undifferentiated, sandy sediments that mark the latest Quaternary sequences of the southeastern Atlantic Coastal Plain. Overall, the eastern study sites display few visual changes in stratigraphy that mark the YDB layer, and thus, the Pt anomaly provides a highly useful tool for locating that boundary, particularly because collecting carbon-rich material for radiocarbon dating is very difficult on the Coastal Plain and because OSL dating typically has larger uncertainties at ~13,000 years ago than for younger sediments. In contrast, the YD onset layer is more easily identified at the four intensively studied and well-dated western and Midwestern YDB study sites that have coeval Pt anomalies and YDB proxies, because the anomalies lie directly beneath distinctive, easily identified “black mat” layers^28^. Recognition of Pt anomalies at the YDB in sites stretching from the Atlantic to the Pacific Ocean verifies a continental-scale horizon marker for the YD onset in North America. Furthermore, the independent discoveries of Pt anomalies in Russia^23^, Lithuania^24^, Belgium, and the Netherlands^25^ suggests that the horizon marker also exists in Europe and Asia, although additional site studies are needed for confirmation.

## Conclusions

Regardless of origin, the consistent presence of anomalous Pt concentrations within sediments from multiple archaeological sites across North America that date to the onset of the YD Chronozone is compelling. This chronostratigraphic marker at the YDB will be useful in geoarchaeological research for inferring the relative stratigraphic position of early Paleoindian (Clovis) and post-Clovis occupations over large portions of North America, especially for sites lacking visual stratigraphy and/or unambiguously stratified, well-dated archaeological assemblages. Regarding limitations of Pt as a chronostratigraphic marker, this study finds that the Pt enrichment is highly variable, may be only slightly above background in some cases, often occurs within narrow (<2.5-cm) sediment intervals, and may be subject to the same post-depositional processes affecting sediments and artifacts (e.g., winnowing, reworking, and pedogenesis).

The same limitations discussed above for Pt also apply to methods of absolute dating of archaeological sites. Radiocarbon and OSL dating rely on carbon and sand grains respectively that are subject to reworking and displacement. In the case of the Pt anomalies reported here, their use as a temporal marker for the onset of the YD is likely to be at least as accurate as OSL dating given the large associated error (±500 years or more) typical for OSL dating of 13,000 year-old sediments. Ideally, the Pt anomaly will be useful for identifying areas to be targeted later for absolute dating.

This study finds no evidence to contradict the conclusions of Petaev *et al*.^1^ that the Greenland Pt enrichment most likely resulted from an extraterrestrial source, whether the Pt originated from the impactor and/or target rocks. In addition, our findings show no contradiction with the Younger Dryas impact hypothesis, although detailed evidence for such an impact or airburst is beyond the scope of this paper.

Our evidence indicates the Pt anomaly is a useful time marker horizon, and the ability to locate this chronostratigraphic marker easily within de-glaciated sediments is a significant finding. This time marker will allow more robust age-depth models and facilitate understanding of the effects of YD climate change on early Paleoindians, flora, and fauna across the Bølling-Allerød/YD transition.

## Methods

In all, 199 bulk sediment samples of ~50 gm each were analyzed for Pt and palladium (Pd). A bulk weight of 50 gm or more is strongly recommended due to potential problems from the nugget effect (see Supplementary Information, “Heterogeneous Distribution of Pt and Pd”). Samples were collected from each site to bracket the onset of the YD Chronozone using existing archaeostratigraphic and/or geochronologic evidence. For western sites, samples were discontinuous and of variable thickness across the defined YDB, while samples from Sheriden Cave in Ohio, and all seven eastern sites, were collected in continuous fashion (Supplemental Tables 3 and 4). Each sample was analyzed using FA and ICP-MS to determine Pt and Pd abundances with a lower detection limit of 0.1 parts per billion (ppb). The accuracy of the lab results was verified with blanks and known standards (Supplementary Information, “Materials and Methods”). In Supplementary Tables 3–6, detailed data are provided for each site related to depositional environment, age, sampling strategy, and earliest cultural occupation.

## Additional Information

**How to cite this article**: Moore, C. R. *et al*. Widespread platinum anomaly documented at the Younger Dryas onset in North American sedimentary sequences. *Sci. Rep.* **7**, 44031; doi: 10.1038/srep44031 (2017).

**Publisher's note:** Springer Nature remains neutral with regard to jurisdictional claims in published maps and institutional affiliations.

## Supplementary Material

Supplementary Information
