# Correction: Widespread platinum anomaly documented at the Younger Dryas onset in North American sedimentary sequences

**DOI:** 10.1038/s41598-026-48937-x

**Published:** 2026-05-06

**Authors:** Christopher R. Moore, Allen West, Malcolm A. LeCompte, Mark J. Brooks, I. Daniel Randolph, Albert C. Goodyear, Terry A. Ferguson, Andrew H. Ivester, James K. Feathers, James P. Kennett, Kenneth B. Tankersley, A. Victor Adedeji, Ted E. Bunch

**Affiliations:** 1 Savannah River Archaeological Research Program, South Carolina Institute of Archaeology and Anthropology, University of South Carolina, P.O. Box 400, New Ellenton, SC 29809 USA; 2 GeoScience Consulting, Dewey, AZ 86327 USA; 3 Center of Excellence in Remote Sensing Education and Research, Elizabeth City State University, Elizabeth City, NC 27921 USA; 4 Department of Anthropology, East Carolina University, Greenville, NC 27858 USA; 5 South Carolina Institute of Archaeology and Anthropology, Columbia, SC 29208 USA; 6 Department of Environmental Studies, Wofford College, 429 N Church Street, Spartanburg, SC 29303-3663 USA; 7 Department of Geosciences, University of West Georgia, 1601 Maple Street, Carrollton, GA 30118 USA; 8 Luminescence Dating Laboratory, University of Washington, 125 Raitt Hall, Seattle, WA 98195-3412 USA; 9 Department of Earth Sciences and Marine Science Institute, University of California, Santa Barbara, USA; 10 Departments of Anthropology and Geology, University of Cincinnati, Cincinnati, OH 45221 USA; 11 Department of Natural Sciences, Elizabeth City State University, Elizabeth City, NC 27909 USA; 12 Geology Program, School of Earth Science and Environmental Sustainability, Northern Arizona University, Flagstaff, AZ 86011 USA

Correction to: *Scientific reports* 10.1038/srep44031, published online 09 March 2017

The original version of the Article contains errors in the reporting and interpretation of the Petaev et al. 2013 (Reference 1) findings. The Authors now mention the correct platinum (Pt) values originally reported in Petaev et al. 2013 and have revised statements accordingly. Contextually relevant References have been added and are listed below as References 2 and 3.

2. Boslough, M. Greenland Pt anomaly may point to noncataclysmic Cape York meteorite entry. *Proc. Natl. Acad. Sci. U.S.A.* **110** (52) E5035-E5035 (2013).

3. Petaev, M. I., Huang, S., Jacobsen, S. B. & Zindler, A. Reply to Boslough: Is Greenland Pt anomaly global or local? *Proc. Natl. Acad. Sci. U.S.A.* **110** (52) E5036-E5036 (2013b).

As a result, in the Introduction section,

“In 2013, Petaev et al.^1^ discovered an anomalously large platinum (Pt) peak in ice core samples from the Greenland Ice Sheet Project 2 (GISP2), thus providing the most compelling evidence to date for a catastrophic extraterrestrial event coincident with the onset of the Younger Dryas (YD) climate episode. In the study by Petaev et al., high-resolution (2.5–4.6 y) time-series of ice core samples were analyzed for trace and major element concentrations using inductively coupled plasma mass spectrometry (ICP-MS). Petaev et al.^1^ reported the presence of a Pt peak anomaly at the Bølling-Allerød/Younger Dryas Boundary (YDB), coincident with a large shift in δ^18^O values, confirming the onset of cooler conditions at the beginning of the YD interval. This peak interval is represented by a rise in Pt concentrations over 14 years and subsequent drop during the following 7 years, consistent with the known residence time of stratospheric dust^1^. This sharply defined Pt anomaly at the YD onset in GISP2 is coeval with other YDB impact-related proxies, including nanodiamonds and melted spherules, found in Greenland and across four continents and is proposed by Petaev et al. to have resulted from a highly fractionated, Ir-deficient, iron-rich, extraterrestrial impactor.”

should read:

“In 2013, Petaev et al.^1^ reported a platinum (Pt) anomaly in ice core samples from the Greenland Ice Sheet Project 2 (GISP2) near the onset of the Younger Dryas (YD) climate episode. In the study by Petaev et al., high-resolution (2.5–4.6 y) time-series of ice core samples were analyzed for trace and major element concentrations using inductively coupled plasma mass spectrometry (ICP-MS). Petaev et al.^1^ reported the presence of a Pt anomaly at the Bølling-Allerød/Younger Dryas Boundary (YDB), broadly coincident with a shift in δ^18^O values marking the onset of cooler conditions. The anomaly spans approximately 21 years, with an average Pt concentration of ~30 parts per trillion (ppt) and a single-sample peak of ~80 ppt (0.08 ppb). Petaev et al. interpreted this anomaly as consistent with atmospheric deposition of Pt-rich dust, possibly including an extraterrestrial contribution, but noted that the source and mechanism could not be identified with certainty^1^. This Pt anomaly at the YD onset in GISP2 is broadly contemporaneous with other proposed YDB impact-related proxies reported from Greenland and from sites on four continents, including nanodiamonds in Greenland and melted spherules at multiple terrestrial sites. Petaev et al. interpreted the Greenland Pt anomaly as potentially consistent with input from an unusual Pt-rich, Ir-deficient extraterrestrial source, although the precise source and mechanism remained uncertain. Interpretations of the Greenland Pt anomaly have been the subject of contemporaneous discussion, including a comment by Boslough (2013)^2^ and a reply by Petaev et al. (2013b)^3^, which highlighted uncertainties regarding the spatial extent, timing, and source of the Pt enrichment.”

In the Results and Discussion section,

“Results that are reported here for 11 sites from six U.S. states, from the Atlantic to Pacific coasts, provide strong evidence for above-background enrichment in Pt within sediments that date to the onset of YD climate change at ~12,800 Cal B.P. (Figs 2 and 3; Supplementary Tables 1 and 2). Pt abundances from our study sites averaged 6.0 ppb (range: 0.3 to 65.6 ppb) compared to background abundances above and below the YDB layer averaging 0.3 ppb. Average background Pt concentrations are all lower than crustal abundance of 0.5 ppb, whereas average YDB concentrations are 12× higher. These concentrations are also higher than the peak Pt concentration (~80 parts per trillion [ppt] or 0.1 ppb) reported at high chronological resolution from the GISP2 ice-core in Greenland by Petaev et al.^1^. All study sites contain significant Pt peaks that are ~3 to 66× higher than in Greenland, possibly because a) Greenland was further from proposed North American impact sites, b) atmospheric circulation from North America carried less Pt to Greenland, c) deposition and preservation of Pt and other impact proxies are highly variable, d) normal Pt concentrations found in sediment add a background level upon which the YDB Pt concentrations are superimposed, e) Greenland ice accumulated at a faster rate than terrestrial sediment, diluting the relative Pt concentrations, and/or f) the lower resolution of this study’s samples may be roughly equivalent to the total input of Pt to Greenland across ~21 years. This wide variation in Pt concentrations among sites is possibly due to post-depositional processes such as winnowing and erosion. In addition, discontinuous sampling at a few sites and the accidental crosscutting of invisible depositional boundaries during sampling means that the highest value of Pt may not have been obtained.”

should read:

“Results that are reported here for 11 sites from six U.S. states, from the Atlantic to Pacific coasts, provide strong evidence for above-background enrichment in Pt within sediments that date to the onset of YD climate change at ~12,800 Cal B.P. (Figs 2 and 3; Supplementary Tables 1 and 2). Pt abundances from our study sites averaged 6.0 ppb (range: 0.3 to 65.6 ppb) compared to background abundances above and below the YDB layer averaging 0.3 ppb. Average background Pt concentrations are all lower than crustal abundance of 0.5 ppb, whereas average YDB concentrations are 12× higher. These concentrations are also higher than both the average Pt concentration (~30 ppt) and the single-sample peak Pt concentration (~80 ppt, or 0.08 ppb) reported at high chronological resolution from the GISP2 ice core in Greenland by Petaev et al.^1^. All study sites contain significant Pt peaks that are substantially higher than those reported from Greenland, possibly because a) Greenland was further from proposed North American impact sites, b) atmospheric circulation from North America carried less Pt to Greenland, c) deposition and preservation of Pt and other impact proxies are highly variable, d) normal Pt concentrations found in sediment add a background level upon which the YDB Pt concentrations are superimposed, e) Greenland ice accumulated at a faster rate than terrestrial sediment, diluting the relative Pt concentrations, and/or f) the lower resolution of this study’s samples may be roughly equivalent to the total input of Pt to Greenland across ~21 years. This wide variation in Pt concentrations among sites is likely influenced by differences in depositional environment, sampling resolution, sedimentation rate, and post-depositional processes. In addition, discontinuous sampling at a few sites and the accidental crosscutting of invisible depositional boundaries during sampling means that the highest value of Pt may not have been obtained.”

and,

“This leaves only atmospheric input through an extraterrestrial impact as the most plausible source of Pt enrichment (Supplementary Information, “Potential Sources of YDB Platinum”).”

should read:

“These observations are consistent with atmospheric input of non-local Pt-enriched material at the YDB. An extraterrestrial contribution remains one possible explanation, but the present study does not independently identify the carrier or ultimate source of the Pt enrichment (Supplementary Information, “Potential Sources of YDB Platinum”).”

Finally, in the Conclusions section,

“This study finds no evidence to contradict the conclusions of Petaev et al.^1^ that the Greenland Pt enrichment most likely resulted from an extraterrestrial source, whether the Pt originated from the impactor and/or target rocks. In addition, our findings show no contradiction with the Younger Dryas impact hypothesis, although detailed evidence for such an impact or airburst is beyond the scope of this paper.”

should read:

“This study finds that anomalous Pt enrichments occur in multiple North American sedimentary sequences near the onset of the Younger Dryas and are broadly consistent with the Greenland Pt anomaly reported by Petaev et al.^1^. Our findings are compatible with interpretations involving a continentally distributed influx of Pt-rich material at the Younger Dryas onset, although they do not independently resolve the ultimate source of the Greenland anomaly or the specific mechanism responsible for Pt enrichment, which lies beyond the scope of this study.”
