# Supplementary material for: Widespread platinum anomaly documented at the Younger Dryas onset in North American sedimentary sequences
Source: Sci Rep. 2017 Mar 9;7:44031. doi: 10.1038/srep44031 (PMC5343653; doi:10.1038/srep44031)
Supplement: Supplementary Information [file srep44031-s1.pdf]

Supplementary Information: ***Widespread platinum anomaly documented at the Younger Dryas onset in North American sedimentary sequences***

Christopher R. Moore,[1]* Allen West,[2] Malcolm A. LeCompte,[3] Mark J. Brooks,[4] I. Randolph Daniel, Jr.,[5] Albert C. Goodyear,[6] Terry A. Ferguson,[7] Andrew H. Ivester,[8] James K. Feathers,[9] James P. Kennett,[10] Kenneth B. Tankersley,[11] A. Victor Adedeji,[12] Ted E. Bunch,[13]

[1]Savannah River Archaeological Research Program, South Carolina Institute of Archaeology and Anthropology, University of South Carolina, P.O. Box 400, New Ellenton, SC 29809, USA. [2]GeoScience Consulting, Dewey, AZ, 86327, USA. [3]Center of Excellence in Remote Sensing Education and Research, Elizabeth City State University, Elizabeth City, NC 27921, USA. [4]Savannah River Archaeological Research Program, South Carolina Institute of Archaeology and Anthropology, University of South Carolina—Retired, 511 Migrant Camp Road, Batesburg, SC 29006, USA. [5]Department of Anthropology, East Carolina University, Greenville, NC 27858, USA. [6]South Carolina Institute of Archaeology and Anthropology, Columbia, SC 29208, USA. [7]Department of Environmental Studies, Wofford College, 429 N Church Street, Spartanburg, SC 29303-3663, USA. [8]Department of Geosciences, University of West Georgia, 1601 Maple Street, Carrollton, GA 30118, USA. [9]University of Washington, Luminescence Dating Laboratory, 125 Raitt Hall, Seattle, WA 98195-3412. [10]Department of Earth Sciences and Marine Science Institute, University of California, Santa Barbara. [11]Departments of Anthropology and Geology, University of Cincinnati, Cincinnati, OH 45221.[12]Department of Natural Sciences, Elizabeth City State University, Elizabeth City, NC 2792. [13]Geology Program, School of Earth Science and Environmental Sustainability, Northern Arizona University, Flagstaff, AZ 86011.

*To whom correspondence should be addressed. E-mail: MOORECR@mailbox.sc.edu

Materials and Methods

At the three western study sites (Blackwater Draw, Murray Springs, and Arlington Canyon), sampling typically involved collection of discontinuous samples of variable thickness based on well-defined and well-dated stratigraphic zones. Sheriden Cave was sampled continuously but at variable increments. For eastern study sites (n=7), samples were also collected in a continuous fashion at 2.5-cm increments from the ground surface to depths exceeding the established or inferred depth of the YDB (Supplementary Figure 1). Given that most of our eastern study sites consist of visually undifferentiated sand, we tested samples through a significant portion of the exposed profile in order to evaluate relative background concentrations of Pt and Pd in sediments of pre- and post-YD age. All sediment samples were collected in the field directly from cleaned archaeological test unit profiles, stored in plastic bags, allowed to air dry over several days, and thoroughly homogenized before sampling for geochemistry. Samples for testing were scooped out with a plastic spoon, weighed, and repackaged in a plastic bag prior to analysis.

Activation Laboratories (Actlabs), using fire-assay (FA) and inductively coupled plasma mass spectrometry (ICP-MS) after Hoffman and Dunn[1], measured the elemental concentrations of sediment samples from all sites. A sample size of approximately 50 grams of sediment is needed for the "1C-Research" analysis performed by Actlabs. Prior to analysis, each sample is mixed with fire assay fluxes (borax, soda ash, silica, litharge) and silver (Ag) added as a collector. The mixture is placed in a crucible and preheated at 850°C, intermediate at 950°C, and finished at 1060°C for a total of 60 minutes. After the crucibles are removed from the assay furnace, the molten slag is poured into a mould leaving a lead button. The lead button is then preheated to 950°C to recover the Ag (doré bead) + Au, Pt and Pd.

The Ag doré bead is digested in hot (95°C) HNO_3 + HCl with a special complexing agent to prevent the Au, Pd, and Pt from adsorbing onto the test tube. After cooling for 2 hours the sample solution is analyzed for Au, Pt, and Pd using a Perkin Elmer Sciex ELAN 9000 ICP-MS. On each tray of 42 samples, there are 2 method blanks, 3 sample duplicates, and 2 certified reference materials. The ICP-MS is recalibrated every 45 samples. Smaller sample splits are used for high chromite or sulfide samples. Measurements are reported in parts per billion (ppb) with a lower limit of detection for Pt at 0.1 ppb. Results are presented in Supplementary Tables 1 and 2.

Study Sites

In the western USA, sites tested include: Arlington Canyon on Santa Rosa Island near Southern California, Murray Springs near Sierra Vista, Arizona, and Blackwater Draw near Clovis, New Mexico. In the Midwest, we tested samples from deeply stratified deposits at Sheriden Cave in Ohio. Sites tested in the eastern USA include: Squires Ridge and Barber Creek on the Tar River in eastern North Carolina, the Kolb Site on the Pee Dee River in northeast South Carolina, Flamingo Bay and Pen Point on the Department of Energy's Savannah River Site (SRS) in South Carolina, and the Topper Site and Johns Bay in southeastern South Carolina (see Figure 1 in main text; Supplementary Tables 3 and 4).

The *Arlington Canyon Site* is located on Santa Rosa Island about 53 km southwest of Santa Barbara in Southern California. The area of study is a 5-m high stream-cut cliff within an alluvial terrace[2]. Detailed stratigraphy for the site is available in Kennett *et al.*[3]. YDB impact proxies are found at the base of the cliff within a 44-cm-thick "black mat" consisting of organic-rich, silty mud[4,5]. The AC-003 profile was sampled to test for the presence of Pt across the YDB as determined by 12 AMS dates[3]. An estimate of 12.8 ka was proposed for the organic rich layers based on linear interpolation. A Pt and Pt/Pd anomaly occurs at the YDB and is associated with a peak in nanodiamonds[4,5] and microspherules[2] (Supplementary Table 2 and Supplementary Figures 2A, 3A, and 4). Kennett *et al.*[3] considered the Arlington Canyon profile (501 to 394 cm) to represent a catchment basin where rapidly deposited YDB fill overlies the primary YDB layer at the bottom, where we find the Pt peak.

Murray Springs is located 10 km east of Sierra Vista, Arizona and consists of alluvium/colluvium, marl, and/or lacustrine mudstone[2]. Within stratum F1, incised marl deposits are filled with stream-channel sands and gravels that are capped with a "carbon-rich" black mat (stratum F2). Silty sediments from colluvial and alluvial deposition, including slopewash, overlie this black mat. Haynes and Huckell[6] determined that stratum F1 at Murray Springs is Clovis age based on the presence of Clovis artifacts and mammoth bones. The black mat at Murray Springs was deposited on top of Unit F1 at the onset of the YD at ~12.8 ka based on a second order polynomial regression of 7 AMS dates at a depth of 2.46 m below surface. Ten discontinuous samples for Pt analysis were collected from this site between 216 and 262 cmbs and identified as stratigraphic units E, F1, and F2[2]. A large Pt and Pt/Pd anomaly occurs at the YDB and is

associated with a peak in nanodiamonds[5] and microspherules[2] (Supplementary Table 2 and Supplementary Figures 2B, 3B, and 5).

Blackwater Draw is about 18 km southeast of Clovis, New Mexico. The site consists of Pleistocene sandy alluvium capped by diatomite and silty muds[2]. Numerous Clovis artifacts and mammoth bones have been found at stratigraphic Unit C with a thin black mat (Unit D) deposited on top[7,8]. The contact between Units C and D represents the YDB. A YDB age determination for this contact was based on logarithmic interpolation of five conventional and AMS radiocarbon dates and provided an estimate of ~12.8 ka[2]. Nine sediment samples were collected from a lithostratigraphic column from inside the South Bank Interpretive Center for Pt analysis. These samples bracket the YDB between Units C and D. A Pt and Pt/Pd anomaly occurs at the YDB and is associated with a peak in nanodiamonds[5,9] and microspherules[2] (Supplementary Table 2 and Supplementary Figures 2C, 3C, and 6).

Sheriden Cave is 4 km northwest of Carey, Ohio. The site consists of deeply buried, stratified "matrix supported gravel" deposits within a collapsed karst cavern[2,10-16]. The YDB layer at Sheriden Cave is defined by the presence of Clovis points and osseous tools within a charcoal-rich layer at ~10.5 m below the floor of the cave. We adopt the chronology established by Redmond and Tankersley[14] and Waters *et al.*[16] based on 29 AMS dates from the cave. Three AMS dates from the YDB layer provide an age (ca. 12.8 ka) consistent with the YDB from other sites[2]. For this study, eight continuous samples of variable increment thickness were collected from an 81-cm-thick sequence from within the cave, including the YDB black-mat layer at ~10.5 m below the original cave floor prior to archaeological excavations. A Pt and Pt/Pd anomaly occurs at the YDB and is associated with a peak in nanodiamonds[9] and microspherules[2] (Supplementary Table 2 and Supplementary Figures 2D, 3D, and 7).

The *Squires Ridge* (31ED365) and *Barber Creek* (31PT259) sites are archaeologically stratified sand ridges on the lower paleo-braidplain terrace of the Tar River in eastern North Carolina. Both sites are multicomponent with stratified sequences from Early Archaic through Woodland. These sites have been the focus of intensive geoarchaeological survey for more than a decade[17-27]. In the upper meter, site formation processes at both Squires Ridge and Barber Creek consisted of periodic source-bordering aeolian sedimentation of medium to fine sands with fluvial contributions from large megaflood events. Fluvial sediments below the aeolian

sediments are likely related to braided river conditions prevalent along the Tar River during the Pleistocene.

For this study, sediment columns from each site were collected as part of an ongoing geoarchaeological survey of the Tar River and were analyzed to determine if samples contained elevated Pt from sediments of likely YD age. This determination is based on site chronologies developed through analysis of archaeostratigraphy and detailed geochronology (both OSL and radiocarbon). Multiple Pt and Pt/Pd anomalies were found at Squires Ridge in sediments of likely YD age (based on OSL dating) and more recent age sediments indicating reworking of Pt-rich sediments at this particular location (Supplementary Table 1 and Supplementary Figures 8A and 9). The presence of multiple Pt anomalies at Squires Ridge, underscores the complex taphonomic processes operating within shallow, stratified, sandy archaeological sites. On the other hand, the Barber Creek Site is a geomorphically similar landform along the same river drainage as Squires Ridge, yet has a single Pt anomaly (Supplementary Table 1 and Supplementary Figures 8B and 10) at a depth consistent with the lower YDB based on archaeostratigraphy, single-grain OSL, and an AMS date from an adjacent excavation unit.

Many sandy sites in the eastern US contain Paleoindian and Early Archaic components within the same stratigraphic zone or with very little separation (e.g., Topper, Kolb, and Flamingo Bay). As a result, Pt anomalies may be expected to occur in some sites within stratigraphic sequences that contain both Paleoindian and Early Archaic artifacts or with Early Archaic artifacts sitting immediately above YD-age sediments. Archaeological occupations at Squires Ridge, beginning with Early Archaic side-notched stone tool industries, are found only within and above the deepest Pt anomaly and only pre-cultural, archaeologically sterile zones lie underneath the deepest Pt anomaly. This is consistent with post-depositional processes and reworking of Pt-enriched sediments during periodic landform aggradation events during and after the YD event.

Previous studies have identified microspherules at Barber Creek from sediments dated with single-grain Optically Stimulated Luminescence (OSL) that overlap (at 2-sigma) with the YDB[2,28]. Based on the presence of microspherules associated with the OSL date, Wittke *et al.*[2] identified the likely depth of the YDB at Barber Creek at 100 cmbs; however, the Pt anomaly (this study) is from 107.5-110 cmbs, consistent with an AMS date (12,860-12,300 Cal. B.P.; *INTCAL04* 2-sigma calibration) on wood charcoal recovered from an adjacent excavation unit

between 100 and 110 cmbs (Supplementary Figures 10 and Supplementary Table 5). The microspherules from Barber Creek were identified based on a cursory examination of three samples associated with the OSL date at 100 cm and may represent aeolian reworking of sediments similar to Squires Ridge. Sediments at the depth of the Pt anomaly have not been analyzed for spherules. Only additional analyses of sediments to look for microspherules will resolve this issue. In any event, the Pt anomaly is a more convincing YDB datum since we tested continuous samples over a large portion of the excavation profile and found only a single Pt anomaly—consistent with what was found in GISP2 ice core at the YDB.

The *Johannes Kolb site* (38DA75) is located in the Great Pee Dee Heritage Preserve in South Carolina and lies within the Middle Coastal Plain portion of the Pee Dee River Valley. The landform sits on the first alluvial terrace overlooking the river. Geomorphically, the Kolb site is overbank sediment (between 2-3 m thick) that was deposited immediately adjacent to the primary channel since the late Pleistocene[29]. Since 1997, the South Carolina Department of Natural Resources (SCDNR) and the South Carolina Heritage Trust Program have conducted archaeological fieldwork at Kolb. Excavations have revealed evidence of intensive occupation with stratified sequences from historic through Paleoindian at depths of up to 1.2 m. Recent geoarchaeological investigations at the Kolb site by the authors have included close-interval sedimentology, analysis of archaeostratigraphy, and single-grain OSL dating. A large Pt and Pt/Pd anomaly (Supplementary Table 1) was found at Kolb between single-grain OSL dates (Supplementary Table 6) that bracket the YDB. Temporally diagnostic artifacts are found in correct stratigraphic order and are consistent with the placement of the lower YDB at ca. 90-92.5 centimeters below surface (cmbs) (Supplementary Figures 8C and 11).

Flamingo Bay (38AK469) is a stratified multicomponent site located on the eastern sand rim of Flamingo Bay, a Carolina bay on the U.S. Department of Energy's (DOE) Savannah River Site (SRS) in the Upper Coastal Plain of South Carolina[30-31]. Clovis points and artifacts have been found in shallowly buried context often conflated with later Early Archaic occupations[32]. Carolina bays are shallow, oriented (NW-SE in the Carolinas), elliptically shaped lakes occurring in large numbers throughout the South Atlantic Coastal Plain[33-37]. Carolina bays often have elevated sand rims composed of fine sand to gravel-sized sediments. Sand rims are paleoshorelines constructed through lacustrine processes involving high-energy shorefaces and eolian sedimentation[30,38,34,39]. Recent excavations at 38AK469 by the Savannah River

Archaeological Research Program (SRARP) have focused on understanding the nature of site burial and taphonomic processes within Carolina bay sand rims through an analysis of archaeological stratigraphy, geophysics, and sediments, as well as the development of an OSL and radiocarbon (^{14}C) geochronology[40].

Conflicting AMS dates and OSL age estimates from Flamingo Bay (38AK469) preclude an accurate assessment of chronostratigraphy; however, two broken Clovis point fragments and numerous Clovis unifacial tools and scrapers[32] at the site occur at ca. 50-55 cmbs in the downslope section of the main excavation block where samples were collected for Pt analysis. The stratigraphic position for Early Paleoindian (i.e., Clovis technocomplex), which dates to ca. 13,250 to 12,850 Cal. B.P.[41], occurs at ~50-55 cmbs in this portion of the excavation block. The large Pt and smaller Pt/Pd anomaly at Flamingo Bay is located at the same depth or just below Clovis artifacts. This depth is consistent with the likely position of the YDB (Supplementary Table 1 and Supplementary Figures 8D and 12). More refined chronological controls are needed at Flamingo Bay before the correlation of Pt with YDB-age sediments can be established with absolute certainty. The data are however, consistent with those reported for virtually all other study sites including thoroughly-dated western and Midwestern sites.

Pen Point (38BR383) is a stratified, multicomponent, overbank alluvial site located in Barnwell County, South Carolina on the U.S. Department of Energy's (DOE) Savannah River Site (SRS). The site is located on the edge of the first (T1A) terrace of the Savannah River at its confluence with Pen Branch, a tributary of the Savannah River. Site formation processes at Pen Point consisted of a series of fining-upward point bar sediments deposited during periodic megaflood events beginning in the late Pleistocene (post LGM) and extending through most of the Holocene[42]. Excavations by the SRARP in the early to mid-1980s recovered over 90,000 artifacts, with clear evidence of archaeological stratigraphy from Woodland through Late Paleoindian contained within more than 1 m of alluvial sand[42]. "Pen Point contains two discrete early to mid-Holocene density modes within a distribution of low-density remains above, below, and between the observed modes[42]." Although the site lacks a radiocarbon chronology and was excavated before the widespread use of luminescence dating, Pen Point revealed a wide-range of temporally diagnostic artifacts in correct chronostratigraphic order, including temporally diagnostic hafted bifaces from Late Paleoindian (Dalton) through Mississippian. In early 2016, a sediment column was acquired from Pen Point by re-excavating one of the old test units and

exposing an intact profile. The age range for Dalton is within the middle to later part of the YD chronozone (ca. 12,500-11,300 Cal. B.P.)[43] and the Pt and Pt/Pd anomalies are stratigraphically deeper, which is consistent with the lower YDB (Supplementary Table 1 and Supplementary Figures 8E and 13).

The *Topper site* (38AL23) is a stratified, multicomponent, prehistoric site located in Allendale County, SC on the banks of the Savannah River. It is a quarry for Coastal Plain chert, and numerous quarry-related sites are associated with it. Clovis is well represented there, as documented by several excavations. Diagnostic Clovis artifacts include broken Clovis points, numerous Clovis biface preforms, and prismatic blades[44-48]. On the Topper site hillslope, Clovis-age artifacts are buried by upwards of 1 m of colluvial/slopewash sand and form a distinctive occupation surface identifiable across much of the site by the presence of dense quarry debris and tools[49]. One Clovis-age radiocarbon date has also been obtained from this occupation surface associated with diagnostic Clovis artifacts[50,44,2]. A small Pt and Pt/Pd anomaly occurs just above the "Clovis Floor" at Topper at a depth of 95-97.5 cmbs and is consistent with the lower YDB at this location (Supplementary Table 1 and Supplementary Figures 8F and 14). A peak in microspherules[2] and nanodiamonds[5] was previously identified at Topper above the Clovis floor within YDB-age sediments; however, those analyses are not available for the excavation unit we tested for Pt and Pd.

Johns Bay (38AL246) is a stratified multicomponent site located on a large Carolina bay (~0.7 km along its long axis and 0.5 km at its widest point). Johns Bay has a prominent eastern sand rim merging laterally into a markedly elevated (~3 m), broad, parabolic dune-shaped landform on the southeastern bay margin. The bay basin is open, characterized by low, herbaceous vegetation and an open-water pool (~0.5 hectares) at the south end. The southeastern sand rim was targeted for geoarchaeological investigations, including detailed sedimentology and single-grain OSL dating. The site is dominated by Archaic period material, with the Early Archaic most prevalent. OSL ages (Supplementary Table 6) are consistent with the archaeology and bracket the YD chronozone between 80 and 100 cmbs. Archaeologically-stratified sequences are present at Johns Bay with buried features, artifact clusters, and temporally diagnostic artifacts in proper chronostratigraphic order. Radiocarbon dates on a buried cultural feature at Johns Bay produced a mid-Holocene age of ca. 7,300 to 7,500 Cal BP, consistent with both the archaeostratigraphy and OSL ages from the site. A small Pt and Pt/Pd anomaly is present at 95-

97.5 cmbs and is consistent with the lower YDB (Supplementary Table 1 and Supplementary Figures 8G and 15).

Summary of PGE Occurrence in the YDB

Pt is one of the platinum group of elements (PGE) that includes iridium (Ir), osmium (Os), ruthenium (Ru), and rhodium (Rh). The following publications have reported peaks in various PGEs in the YDB layer on three continents. Although some references are to conference abstracts that have not been peer-reviewed, they are included because this Pt discovery is novel, and these abstracts add important information that may encourage further research.

Petaev *et al.*[51] analyzed only very small samples of ice (a few cm^3) from a single Greenland ice core and used those to infer deposition rates across the entire Northern Hemisphere. Those authors concluded that the source of the Pt anomaly and Pt/Ir and Pt/Al ratios may have been a very unusual and highly-fractionated, iron-rich, extraterrestrial impactor. However, extrapolating from a small dataset acquired from a single site involves high uncertainties. Various other studies detailed below present evidence for higher concentrations of Pt and Ir at many YDB-age sites, suggesting that the source, whether or not it was an impactor, may not have been highly fractionated. One possible explanation for these differences is that variable sample sizes and rates of deposition, preservation, and fractionation have all affected the relative abundances of PGEs at the various sites. If so, then the exact nature of the PGE source is currently unclear, and more research is required.

Firestone *et al.*[52] reported elevated concentrations of the PGE iridium (Ir) at 11 YDB sites, including Murray Springs, AZ and Blackwater Draw, NM, both reported in this paper to contain high concentrations of Pt.

Beets *et al.*[53] (page 1) reported finding Os at Lommel, Belgium, which generally varies in direct proportion to Ir. The Os occurred in *"a discrete pulse at 12,893 cal yr BP. The observation of the non-radiogenic Os isotope composition would therefore be consistent*

with a meteorite impact." They found the Os peak at the same YDB site and in the same stratum that Firestone reported to contain Ir.

Sharma *et al.*[54] reported YDB-aged extraterrestrial Os: *"We infer that the Central Pacific was a site of deposition of Os resulting from dust cloud following a meteorite impact at 12 ± 4 ka,"* an age that overlaps the YDB with large uncertainties.

Paquay *et al.*[55] (page 3) reported finding small Ir and Pt peaks in the 12,800-year-old YDB layer at Murray Springs, AZ and Lake Hind, AB, Canada at the same YDB stratigraphic levels as peaks in nanodiamonds, magnetic grains, microspherules, and Pt. However, they dismissed the anomalies by stating that *"slightly elevated values are not outside the range known to result from natural authigenic enrichment of Os and Ir …"* However, they offered no evidence to refute an extraterrestrial connection. Our results confirm those of Firestone *et al.*[52] and contradict interpretations by Paquay *et al.*[55].

Haynes *et al.*[56,57] reported high concentrations of Ir at Murray Springs in Arizona ranging from 31-64 ppb in two magnetic fractions from across the YDB. Their values are as much as 32× higher than Firestone reported (2 ppb) but within the range of values at other YDB sites and >3000× terrestrial abundance (0.021 ppb). Their Ir measurements contradict those of Paquay *et al.*[55] who observed smaller Ir anomalies at Murray Springs. When referring to the results of Firestone *et al.*, Haynes *et al.* stated that *"… our analytical data are consistent with their data…"* and *"…neither do our data preclude such an [ET] event."* Inexplicably, Haynes speculated that the Ir levels they found are normal, even though they are anomalously high at >3000× crustal abundance.

Haynes *et al.* also measured 72 ppb of Ir in Curry Draw streambed magnetic grains, which they speculated were background levels of Ir; however, this value is >3000× higher than terrestrial abundance and almost identical to their Murray Springs YDB values. Since the streambed cuts directly through the Ir-enriched YDB layer, the most likely explanation is that the streambed sample contains Ir-rich YDB material that was reworked during streambed erosion.

Mahaney *et al.*[58] (page 10) reported that *"analysis confirms the presence of trace amounts of platinum group metals [ruthenium and rhodium]...* in the YDB in Venezuela, … *with a*

frequency higher than chance occurrence." Mahaney *et al.*[58] (page 1) concludes that *"new evidence … point tentatively to either an asteroid or comet event that reached far into South America."*

Marshall[59] reports finding *"exceptional iridium concentrations"* of 300% of crustal abundance in the YDB layer in southwest England.

Wu *et al.*[60] reported unusually high Os peaks in YDB materials from the Melrose site in PA and the Newtonville (UP) site in New Jersey.

Andronikov *et al.*[61] reported anomalous enrichments in rare earth elements (REE) in YDB sediments from North America and Europe and "*overall higher concentrations of both Os and Ir*" that could "*support the hypothesis that an impact occurred shortly before the beginning of the YD cooling 12.9 ka.*"

Andronikov *et al.*[62] analyzed magnetic microspherules from the YDB layer at Blackwater Draw using scanning electron microscopy (SEM), electron probe microanalysis (EPMA), X-ray diffraction (XRD), and laser-ablation inductively coupled-plasma mass spectrometry (LA-ICP-MS). They report microspherules with melted, dentritic textures (confirmed through a combination of SEM and energy dispersive spectroscopy [EDS]) with very high Pt abundance (18.2 to 460 ppb). Microspherules highly-enriched in Pt from the YDB layer at Blackwater Draw are likely contributing to the Pt anomaly reported in this study from an analysis of bulk sediments.

Andronikov *et al.*[63] examined four sequences from three sites in the Netherlands and Belgium and found elevated Pt and Ir along with other trace elements in the lower Younger Dryas Boundary.

Potential Sources of YDB Platinum

To test potential sources of the YDB Pt enrichments, we compiled data from 766 samples recorded in the Geochemical Earth Reference Model Reservoir Database (GERM) https://earthref.org/GERMRD/[64].

VOLCANOES. To test volcanism as a potential source of YDB Pt enrichment, we compiled data from the GERM Database and found that 24 samples of magma contained Pt ranging from 11.1 to 1.3 ppb (avg: 5.3) with Pt/Pd ratios from 1.5 to 0.8 (Supplementary Table 7). By comparison, Pt concentrations for YDB sites reported in this paper range from 65.6 to 0.3 ppb (avg: 6.0), roughly matching the average Pt concentration of magma and with the range of magmatic Pt falling within the range of Pt concentrations at YDB sites. However, previous work by Firestone *et al.*[51], Bunch *et al.*[65], LeCompte *et al.*[66], Wittke *et al.*[2], in which extensive analyses (SEM/EDX, INAA, PGAA) of sediment, magnetic grains, and magnetic spherules were conducted, showed none of the geochemical characteristics indicative of volcanism. Thus, because the YDB samples contain no detectable magmatic material (~0%), compared to volcanic samples with 100% tephra, it is implausible that YDB samples are volcanic in origin. Furthermore, at Flamingo Bay, which is more than 2000 km away from the closest known volcano, the Pt concentration of 65.6 ppb is higher than that of any known magmatic sample from any volcano in the world, again making volcanism an unlikely source.

In addition, we analyzed Pt concentrations for tephra/ash from five volcanoes, one each in Alaska, Hawaii, Kamchatka, Russia, and Germany (Supplementary Table 10). The one in Germany is from the Laacher See eruption that is generally considered to predate the YD onset by several hundred years, although some propose it to be coeval. In addition, we analyzed pumice from the Akita Prefecture, Japan. Because of prevailing wind direction, all volcanoes, except for Laacher See, could have contributed ash to the Greenland ice sheet. These examples are representative of two main types: island arc volcanoes, e.g., in Japan, and plume volcanoes, e.g., in Hawaii.

Four of the five volcanic samples contained no detectable Pt, meaning that they are unlikely sources of YDB Pt. Only the sample from Diamond Head in Oahu contained high Pt (3.5 ppb), but that sample is composed of 100% tephra/ash. Because YDB sediment contains no detectible tephra (~0%), these volcanoes are an implausible source of the observed concentrations of Pt in YDB Pt sediment. In support of a non-volcanic source of YDB Pt, Gabrielli *et al.*[67] investigated Pt levels in the Greenland ice sheet resulting from recent major eruptions of the volcanoes Pinatubo in the Philippines, the largest in a century, and Hekla in Iceland. The maximum Pt enrichment was 0.00009 ppb, ~100,000× less than most YDB values, making these volcanoes an unlikely source of YDB Pt.

In addition, no volcanogenic samples investigated contain detectable magnetic or glassy spherules, meaning that they are an improbable source of those materials in YDB sediments. In particular, the Laacher See eruption, which is nearly contemporary with the YDB event, lacks detectable spherules and Pt, making it an unlikely source of these YDB proxies.

EARTH'S MANTLE. We also compiled data for 489 samples from the GERM Database[64] to test mantle material as a potential source of YDB Pt. The samples came from ocean trenches, mid-ocean ridge basalt (MORB), ultramafic rocks, and cratons on several continents. Pt concentrations ranged from 13,690 to 0.2 ppb (avg: 2072 to 5.3), making them a potential source of YDB Pt. Even though these concentrations are high, the distribution of Pt from mantle material into the YDB layer widely across Greenland, North America, and NW Asia is implausible through any normal terrestrial process. It is possible, however, that a cosmic impact at the YD onset ejected those materials from target rocks, such as mantle material (cratonic rocks) in Quebec, Canada, as proposed by Wu *et al.*[60]. If so, then the Pt enrichment is impact-related and only indirectly related to Earth's mantle.

METEORITES. We compiled geochemical data for 167 meteorites, including chondrites, achondrites, irons, and urelites, with Pt abundances ranging from 39,300 to 0.2 ppb (avg: 16,077 to 1198 ppb), making all four classes of meteorites possible sources of YDB Pt enrichment. There is another unusual possible source for YDB Pt: meteorites that fell onto the Laurentide Ice Sheet and became trapped in glacial ice during the preceding ~130,000 years. If the YDB impactor struck the ice sheet in eastern Canada, as proposed by Wu *et al.*[60], then based on known flux of meteoritic material, tons of previously fallen meteorites may have been re-melted and ejected, possibly accounting for some YDB Pt enrichment.

IMPACTITES. If a Pt-rich meteorite or comet impacted Earth, the target rocks would have become a melted mix of meteoritic and terrestrial material, and so, should be Pt-enriched. We compiled geochemical data for 86 examples of impactites from three major impact layers at 2.55 Ga, 145, Ma, and 65 Ma. Pt abundances ranging from 380 to 0.6 ppb (avg: 33.2 to 18.3 ppb). This range includes all values found in the Pt-rich YDB layers, meaning that it is possible that the YDB Pt enrichments are due to ejecta from a cosmic impact at 12.8 ka.

Heterogeneous Distribution of Pt and Pd

For some samples, duplicate analyses were performed—both for samples with very high Pt values (i.e., Flamingo Bay) and relatively low ones (i.e., Kolb) in the first test (see Supplementary Tables 1 and 2). This testing has revealed a "nugget effect" with regard to Pt and Pd abundances and indicates that these elements are heterogeneously distributed within the sediment samples. This variation is common when measuring minerals that are present at very low concentrations (ppb). A nugget effect also was previously noted by Firestone *et al.*[52] for another PGE, Ir.

Relative Pt and Pd abundances in duplicate test samples also are variable, suggesting that both Pt and Pd abundances do not vary proportionately in a given sample. Differences in depositional environment, pedogenesis, variable preservation, and variability in the size and elemental composition of source particles, are all likely explanations for the heterogeneous nature of Pt and Pd in sediments. Despite the apparent heterogeneous nature of Pt and Pd, virtually all sites tested produced single Pt and Pt/Pd anomalies within sediments that correspond to the YD onset only. At Flamingo Bay, the original and duplicate tests were significantly different, but both produced Pt anomalies that were an order of magnitude higher than background. At Kolb, tests of YDB age samples originally produced near background level Pt and a small Pd anomaly. A duplicate test revealed a large Pt anomaly consistent with data from other sites. Furthermore, reassay of samples both above and below the Pt anomaly at Flamingo Bay and Kolb were consistent with background values. Differences, including measurements of <0.1 ppb for some reassay samples above and below the Pt anomaly could be due to the use of remaining sample splits that were less than the desired 50 g.

Optically Stimulated Luminescence (OSL)

OSL dating was performed by the University of Washington at Kolb, Squires Ridge, Flamingo Bay, Barber Creek and Johns Bay. Samples were collected in light tight containers from exposed profiles. The sandy quartz-rich sediments were processed following normal procedures to obtain the 180-212µm quartz fraction for luminescence measurements. Measurements in ultra-violet emission were made on single grains using for stimulation a 532nm laser on either a Risø DA-15 or DA-20 instrument. Equivalent dose was obtained by the single aliquot regeneration (SAR) method[68]. Grains were screened for acceptable values using various

standard rejection criteria. Dose rate was measured on bulk sediments using thick source alpha counting, beta counting, and flame photometry. Water content was estimated at 6 ± 3 %. Dose rates, equivalent dose values from various age models, over-dispersion, and age estimates are given in Supplementary Table 6.

Sandy sediments are often of mixed-age because of post-depositional disturbances, commonly caused by burrowing animals. The distribution of equivalent dose among grains for most of these samples had relatively high over-dispersion (scatter that cannot be accounted for by differential precision), higher than that obtained when performing dose recovery. Dose recovery is a test of procedures whereby grains are given a known dose, consistent with a single age. Over-dispersion from dose recovery is intrinsic to the sample. Anything higher in the natural distributions reflects, for the most part, mixing of some kind. OSL uses various age models[69] to interpret single-grain equivalent dose distributions and to make an estimate of the depositional age. For most of the samples, the distributions tended to cluster around either the central tendency (from the central age model) or the largest component of the finite mixture model, with only a handful of older or younger grains mixed in. These estimations are consistent with other data. For Johns Bay, however, ages from the central age model are much too old when compared to other data. In some cases, the presence of partially-bleached grains can contribute an inherited age within sedimentary sequences that mask the true burial age. In addition, Rink *et al.*[70] experimentally demonstrated that ants often disproportionally move sand grains up the soil profile, introducing 'older' grains into more recent age sediments. Both of these processes produce central age model estimates that overestimate the true burial age of the sediments. Application of the minimum age model is common and appropriate in many sandy depositional settings (Feathers *et al.*[71]). At Johns Bay, the use of the minimum age model provided age estimates consistent with radiocarbon dates and archaeostratigraphic information.

References

1. Hoffman, E. L. & Dunn, B., Sample preparation and bulk analytical methods for PGE. CIM Special Volume 54: The Geology, Geochemistry and Mineral Beneficiation of Platinum Group Elements. Edited by Louis J. Cabri, pp.1–11 (2002).

2. Wittke, J. H. *et al*. Evidence for deposition of 10 million tonnes of impact spherules across four continents 12,800 y ago. *Proc. Natl. Acad. Sci. U.S.A.***110**:1–10 (2013).

3. Kennett, D. J. *et al.* Wildfire and abrupt ecosystem disruption on California's northern channel islands at the Ållerød-Younger Dryas boundary (13.0–12.9 ka). *Quaternary Science Reviews* **27**:2530-2545 (2008).

4. Kennett, D. J. *et al.* Shock-synthesized hexagonal diamonds in Younger Dryas boundary sediments. *Proc. Natl. Acad. Sci. U.S.A.***106**(31):12623–12628 (2009a).

5. Kennett, D. J. *et al.* Nanodiamonds in the Younger Dryas boundary sediment layer. *Science* **323**(5910):94 (2009b).

6. Haynes, C. V. Appendix B: Nature and origin of the Black Mat, Stratum F2. *Murray Springs: A Clovis Site with Multiple Activity Areas in the San Pedro Valley, Arizona,* eds. Haynes, C. V., Jr., Huckell, B. B. (University of Arizona Press, Tucson, AZ), pp. 240-249 (2007).

7. Haynes, C. V. Jr. *et al.* A Clovis well at the type site 11,500 B.C.: The oldest prehistoric well in America. *Geoarchaeology* **14**(5):455–470 (1999).

8. Haynes, C. V. Younger Dryas "black mats" and the Rancholabrean termination in North America. *Proc. Natl. Acad. Sci. U.S.A.***105**(18):6520–6525 (2008).

9. Kinzie, C. R. *et al.* Nanodiamond-rich layer across three continents consistent with major cosmic impact at 12,800 Cal BP. *The Journal of Geology* **122**:475–506 (2014).

10. Tankersley, K. B. Sheriden: A Clovis cave site in eastern North America. *Geoarchaeology* **12**(6):713–724 (1997).

10. Tankersley, K. B. Sheriden: A stratified Pleistocene-Holocene cave site in the Great Lakes region of North America. *BAR International Series* **800**:67–75 (1999).

11. Tankersley, K. B. & Redmond, B. G. Fluoride/radiocarbon dating of late Pleistocene bone from Sheriden Cave, Ohio. *Current Research in the Pleistocene* **16**:107–108 (1999a).

12. Tankersley, K. B. & Redmond, B. G. Radiocarbon dating of a projectile point from Sheriden Cave, Ohio. *Current Research in the Pleistocene* **16**:76–77 (1999b).

13. Redmond, B. G. & Tankersley, K. B. Species response to the theorized Clovis comet impact at Sheriden Cave, Ohio. *Current Research in the Pleistocene* **28**:141–143 (2011).

14. Redmond, B. G. & Tankersley, K. B. Evidence of early Paleoindian bone modification and use at the Sheriden Cave site (33WY252), Wyandot County, Ohio. *American Antiquity* **70**(3):503–526 (2005).

15. Tankersley, K. B. & Landefeld, C. S. Geochronology of Sheriden Cave, Ohio: The 1997 field season. *Current Research in the Pleistocene* **15**:136–138 (1998).

16. Waters, M. R., Stafford, T.W. Jr., Redmond, B. G., & Tankersley, K. B. The age of the Paleoindian assemblage at Sheriden Cave, Ohio. *American Antiquity* **74**:107–111 (2009).

17. Barbour, T. E. *Reconstructing the culture history of the multicomponent site Squires Ridge (31ED365) within the northern Coastal Plain of North Carolina.* (Thesis, Department of Anthropology, East Carolina University 2015).

18. Choate, B. C. *Investigations at Barber Creek (31PT259): reconstructing the culture-history of a multicomponent site in the North Carolina Coastal Plain* (Thesis, Department of Anthropology, East Carolina University 2011).

19. Daniel, I. R, Jr. Stratified Early-Middle Holocene remains in the North Carolina Coastal Plain. *Southeastern Archaeological Conference Special Publication* **7**:6–11 (2002a).

20. Daniel, I. R., Jr. *Geoarchaeological investigations at Barber Creek (31PT259), Greenville, North Carolina.* Report prepared under a Historic Preservation Fund Grant from the United States Department of Interior and administered by the North Carolina Division of Archives and History (2002b).

21. Daniel, I. R. Jr., Seramur, K. C, Potts, T. L. & Jorgenson, M. W. Searching a sand dune: shovel testing the Barber Creek Site. *North Carolina Archaeology* **57**:50–77 (2008).

22. Daniel, I. R., Jr., Moore, C. R. & Canyor, E. C. Sifting the sands of time: geoarchaeology, culture chronology, and climate change at Squires Ridge, northeastern North Carolina. *Southeastern Archaeology* **32**:253–270 (2013).

23. McFadden, P. S. *Geoarchaeological investigations of dune formation and artifact deposition at Barber Creek (31PT259) (*Thesis, Department of Anthropology, East Carolina University 2009).

24. Moore, C. R. *Late Quaternary geoarchaeology and geochronology of stratified eolian deposits, Tar River, North Carolina* (Dissertation, Coastal Resources Management Ph.D. Program, East Carolina University 2009).

25. Moore, C. R. & Daniel, I. R. Jr. *Geoarchaeological investigations of stratified sand ridges along the Tar River, North Carolina.* In The Archaeology of North Carolina: Three Archaeological Symposia, edited by Charles R. Ewen, Thomas R. Whyte, and R. P. Stephen Davis, Jr., pp. 1–42. Publication 30. North Carolina Archaeological Council, Raleigh (2011). http://www.rla.unc.edu/NCAC/Publications/NCAC_30.pdf

26. Seramur, K. C. & Cowan, E. A. Geoarchaeology of site 31PT259 at the confluence of Barber Creek and the Tar River Pitt County, Greenville, North Carolina. Report prepared for I.

Randolph Daniel, Jr., Department of Anthropology, East Carolina University, Greenville, North Carolina (2002).

27. Seramur, K. C., Cowan, E. A., Hettinger, D. J. & Daniel, I. R., Jr. Interpreting site formation processes at a stratified archaeology site in a sand dune on the Atlantic Coastal Plain. Paper presented at the 33rd Annual Meeting of the Middle Atlantic Archaeological Conference, Virginia Beach, VA (2003).

28. Kennett, J. P. *et al*. Bayesian chronological analyses consistent with synchronous age of 12,835–12,735 Cal B.P. for Younger Dryas boundary on four continents. *Proc. Natl. Acad. Sci. U.S.A.***112**: E4344–E4353 (2015).

29. Leigh, D. S. Geomorphology of the Kolb Site. Report prepared for Diachronic Research Foundation (2001).

30. Brooks, M. J., Taylor, B. E. & Grant, J. A. Carolina bay geoarchaeology and Holocene landscape evolution on the Upper Coastal Plain of South Carolina. *Geoarchaeology* **11**:481–504 (1996).

31. Brooks, M. J., Taylor, B. E. & Ivester, A. H. Carolina bays: time capsules of culture and climate change. *Southeastern Archaeology* **29**:146–163 (2010).

32. Moore, C. R. & Brooks, M. J. An *in-situ* Clovis assemblage from a Carolina bay sand rim, Aiken County, South Carolina. *South Carolina Antiquities*, **44**:110–112 (2012).

33. Johnson, D. W. The origin of the Carolina bays. New York: Columbia University Press (1942).

34. Kaczorowski, R. T. *The Carolina bays: a comparison with modern oriented lakes.* Technical Report No. 13-CRD, Coastal Research Division, Department of Geology, University of South Carolina, Columbia (1977).

35. Prouty, W. F. Carolina bays and their origin. *Geological Society of America Bulletin* **63**:167–224 (1952).

36. Raisz, E. Rounded lakes and lagoons of the Coastal Plains of Massachusetts. *Journal of Geology* **2**:839–848 (1934).

37. Thom, B. G. Carolina bays in Horry and Marion counties, South Carolina. *Geological Society of America Bulletin* **81**:783–814 (1970).

38. Grant, J. A., Brooks, M. J. & Taylor, B. E. New constraints on the evolution of Carolina bays from ground-penetrating radar. *Geomorphology* **22**:325–345 (1998).

39. Moore, C. R. *et al.* The Quaternary evolution of Herndon Bay: implications for paleoclimate and oriented lake genesis. *Southeastern Geology* **51**:145–171 (2016).

40. Moore, C. R., Brooks, M. J., Ivester, A. H., Ferguson, T. A. & Feathers, J. K. Radiocarbon and luminescence dating at Flamingo Bay (38AK469): implications for site formation processes and artifact burial at a Carolina bay. *Legacy* **16**:16–21 (2012).

41. Waters, M. R. & Stafford, T.W. Jr. Redefining the age of Clovis: implications for the peopling of the Americas. *Science* **315**:122–1126 (2007).

42. Brooks, M. J. & Sassaman, K. E. Point bar geoarchaeology in the Upper Coastal Plain of the Savannah River Valley, South Carolina: a case study. In *Archaeological Geology of North America*, edited by N. P. Lasca and J. E. Donahue, pp. 183-197. Geological Society of America, Centennial Special Volume 4. Boulder, Colorado (1990).

43. Sherwood, S. C., Driskell, B., Randall, A. & Meeks, S. C. Chronology and stratigraphy at Dust Cave, Alabama. *American Antiquity* **69**(3):533–554 (2004).

44. Anderson, D. T., Smallwood, A. M., Goodyear, A. C. & Walters, S. E. The Paleoindian and Early Archaic hilltop occupations at the Topper Site. *Tennessee Archaeology* 8:108–113 (2016).

45. Goodyear, A. C. & Steffy, K. Evidence of a Clovis occupation at the Topper Site, 38AL23, Allendale County, South Carolina. *Current Research in the Pleistocene* **20**:23–25 (2003).

46. Smallwood, A. M. Clovis biface technology at the Topper Site, South Carolina: evidence for variation and technological flexibility. *Journal of Archaeological Science* **37**:2413–2425 (2010).

47. Miller, D. S. Clovis excavations at Topper 2005-2007: examining site formation process at an upland Paleoindian site along the Middle Savannah River. Occasional Papers 1. Southeastern Paleoamerican Survey, South Carolina Institute of Archaeology and Anthropology, University of South Carolina, Columbia (2010).

48. Sain, D. A. Clovis blade technology at the Topper Site (38AL23), assessing lithic attribute variation and regional patterns of technological organization. Occasional Papers No. 2, Southeastern Paleoamerican Survey, South Carolina Institute of Archaeology and Anthropology, University of South Carolina (2012).

49. Waters, M. R., Forman, S. L., Stafford, T. W. Jr. & Foss, J. Geoarchaeological Investigations at the Topper and Big Pine Sites, Allendale County, Central Savannah River, South Carolina. *Journal of Archaeological Science* **36**:1300–1311 (2009).

50. Goodyear, A. C. Update on the 2012-2013 activities of the Southeastern Paleoamerican Survey. *Legacy* **17**:10–12 (2013).

51. Petaev, M., Huang, S., Jacobsen, S. B. & Zindler, A. Large Pt anomaly in the Greenland ice core points to a cataclysm at the onset of Younger Dryas, *Proc. Natl. Acad. Sci. U.S.A.***110**:12917–12920 (2013).

52. Firestone R. B. *et al.* Evidence for an extraterrestrial impact 12,900 years ago that contributed to the megafaunal extinctions and the Younger Dryas cooling. *Proc. Natl. Acad. Sci. U.S.A.* **104**:16016 (2007).

53. Beets, C., Sharma, M., Kasse, K. & Bohncke, S. Search for extraterrestrial osmium at the Allerod -Younger Dryas boundary. American Geophysical Union, Fall Meeting 2008, abstract #V53A-2150 (2008).

54. Sharma, M., Chen, C., Jackson, B. P. & Abouchami, W. High resolution Osmium isotopes in deep-sea ferromanganese crusts reveal a large meteorite impact in the Central Pacific at 12 ± 4 ka. American Geophysical Union, Fall Meeting 2009, abstract #PP33B-06 (2009).

55. Paquay, F. S. *et al.* Absence of geochemical evidence for an impact event at the Bølling–Allerød/Younger Dryas transition. *Proc. Natl. Acad. Sci. U.S.A.***106:**51 21505-21510 (2009).

56. Haynes C. V., Jr. *et al.* The Murray Springs Clovis site, Pleistocene extinction, and the question of extraterrestrial impact. *Proc. Natl. Acad. Sci. U.S.A.***107**(9):4010–4015 (2010a).

57. Haynes, C. V., Jr, Lauretta, D. S. & Ballenger, J. A. M. Reply to Firestone et al.: No confirmation of impact at the lower Younger Dryas boundary at Murray Springs, AZ. *Proc. Natl. Acad. Sci. U.S.A.***107**, pp. E106–E106 (2010b).

58. Mahaney, W. C. *et al.* Evidence from the northwestern Venezuelan Andes for extraterrestrial impact: The black mat enigma. Geomorphology: **116**, iss. 1–2, p. 48–57 (2010).

59. Marshall, W., Head, K., Clough, R. & Fisher, A. Exceptional iridium concentrations found at the Allerød-Younger Dryas transition in sediments from Bodmin Moor in southwest England. Paper #2641, XVIII INQUA-Congress, 21–27 July 2011 in Bern, Switzerland (2011).

60. Wu, Y., Sharma, M., LeCompte, M. A., Demitroff, M. & Landis, J. Origin and provenance of spherules and magnetic grains at the Younger Dryas boundary. *Proc. Natl. Acad. Sci. U.S.A.***110**:38 E3557-3566 (2013).

61. Andronikov, A. V., Lauretta, D. S., Andronikva, I. E. & Maxwell, R. J. On the possibility of a late Pleistocene, extraterrestrial impact: LA-ICP-MS analysis of the Black Mat and Usselo Horizon samples, Abstract for a poster presented at the 74th Meteoritical Society Meeting, held in London U.K., August 8–12 (2011).

62. Andronikov, A. V., Andronikova, I. E. & Loehn, C. W. *et al.* Implications from chemical, structural and mineralogical studies of magnetic microspherules from around the lower Younger Dryas Boundary (New Mexico, USA), Swedish Society for Anthropology and Geography DOI:10.1111/geoa.1212, (2016).

63. Andronikov, A. V., Van Hoesel, A., Andronikova, I. E. & Hoek, W. Z. Trace element distribution and implications in sediments across the Allerød -Younger Dryas in the Netherlands

and Belgium. Geografiska Annaler: Series A, *Physical Geography*, **98**:325-345. DOI:10.1111/geoa.12140 (2016).

64. GERM (Geochemical Earth Reference Model) Reservoir Database for Pt at https://earthref.org/GERMRD/

65. Bunch T. E. *et al.* Very high-temperature impact melt products as evidence for cosmic airbursts and impacts 12,900 years ago. Proc Natl. Acad. Sci. USA **109**(28): E1903–E1912 (2012).

66. LeCompte, M. A. *et al.* Independent evaluation of conflicting microspherule results from different investigations of the Younger Dryas impact hypothesis. Proc. Natl. Acad. Sci. USA 2012; **109**(44):E2960–E2969. Doi 10.1073/pnas.1208603109 PMID: 22988071 (2012).

67. Gabrielli, P. *et al.* Siderophile metal fallout to Greenland from the 1991 winter eruption of Hekla (Iceland) and during the global atmospheric perturbation of Pinatubo. *Chemical Geology* **255**:78–86 (2008).

68. Wintle, A. G. & Murray, A. S. A review of quartz optically stimulated luminescence characteristics and their relevance in single-aliquot regeneration dating protocols. *Radiation Measurements* **41**:369–391 (2006).

69. Galbraith, R. F. *et al.* Optical dating of single and multiple grains of quartz from Jinmium Rock Shelter, Northern Australia: Part I, experimental design and statistical models. *Archaeometry* **41**:339–364 (1999).

70. Rink, J. W. *et al*. Subterranean transport and deposition of quartz by ants in sandy sites relevant to age overestimation in optical luminescence dating. *Journal of Archaeological Science* **40**:2217–2226 (2013).

71. Feathers, J. K. *et al*. Luminescence dating of sand deposits related to Late Pleistocene human occupation at the Cactus Hill Site, Virginia, USA. *Quaternary Geochronology* **1**:167–187 (2006).

72. Kennett, J. P. *et al*. Bayesian chronological analyses consistent with synchronous age of 12,835–12,735 Cal B.P. for Younger Dryas boundary on four continents. *Proc. Natl. Acad. Sci. U.S.A.***112**: E4344-E4353 (2015).

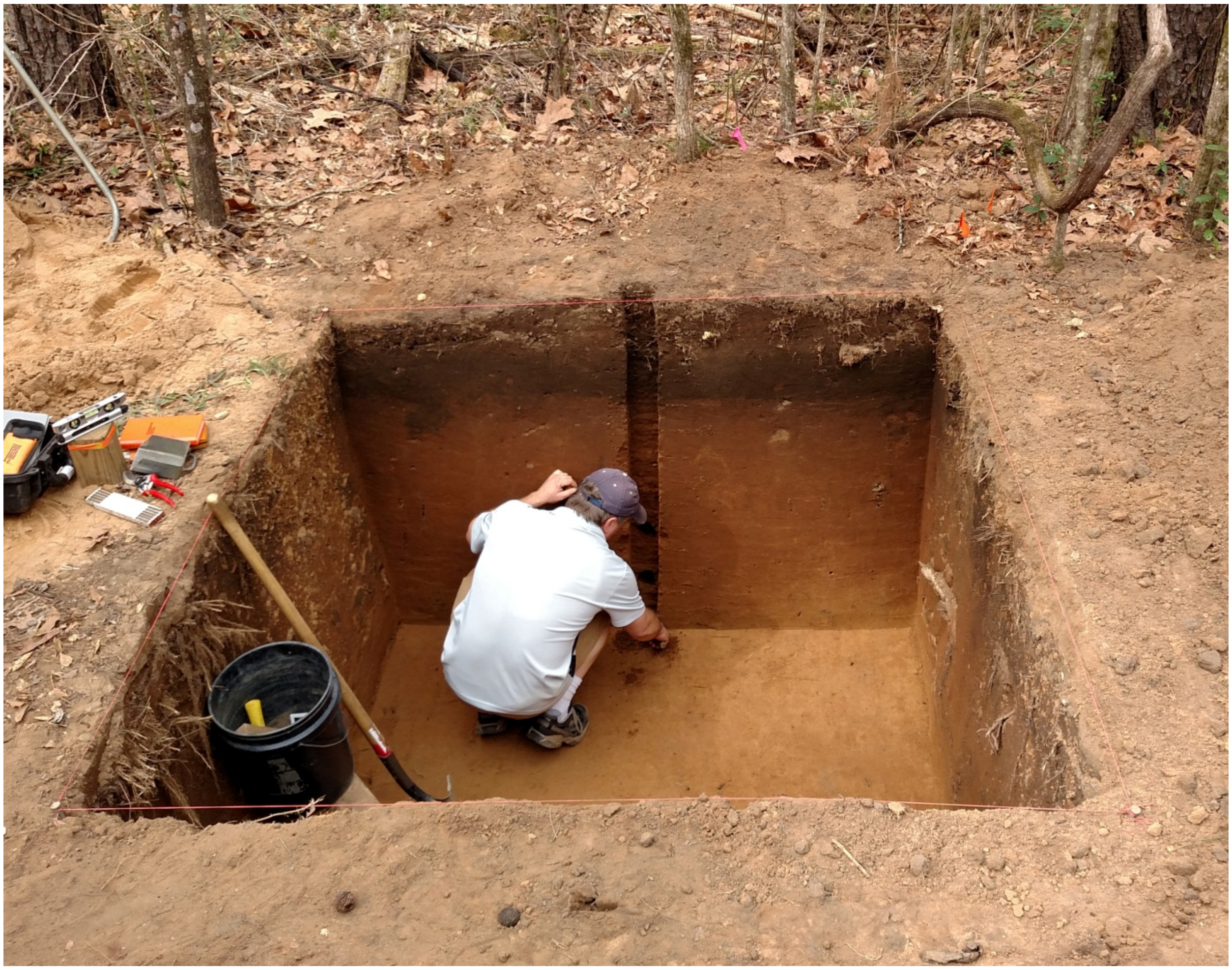

Supplementary Figure 1. Collecting a continuous sediment column in 2.5-cm increments for Pt analysis (Kolb Site, 38DA75 on the Pee Dee River in northeastern South Carolina).

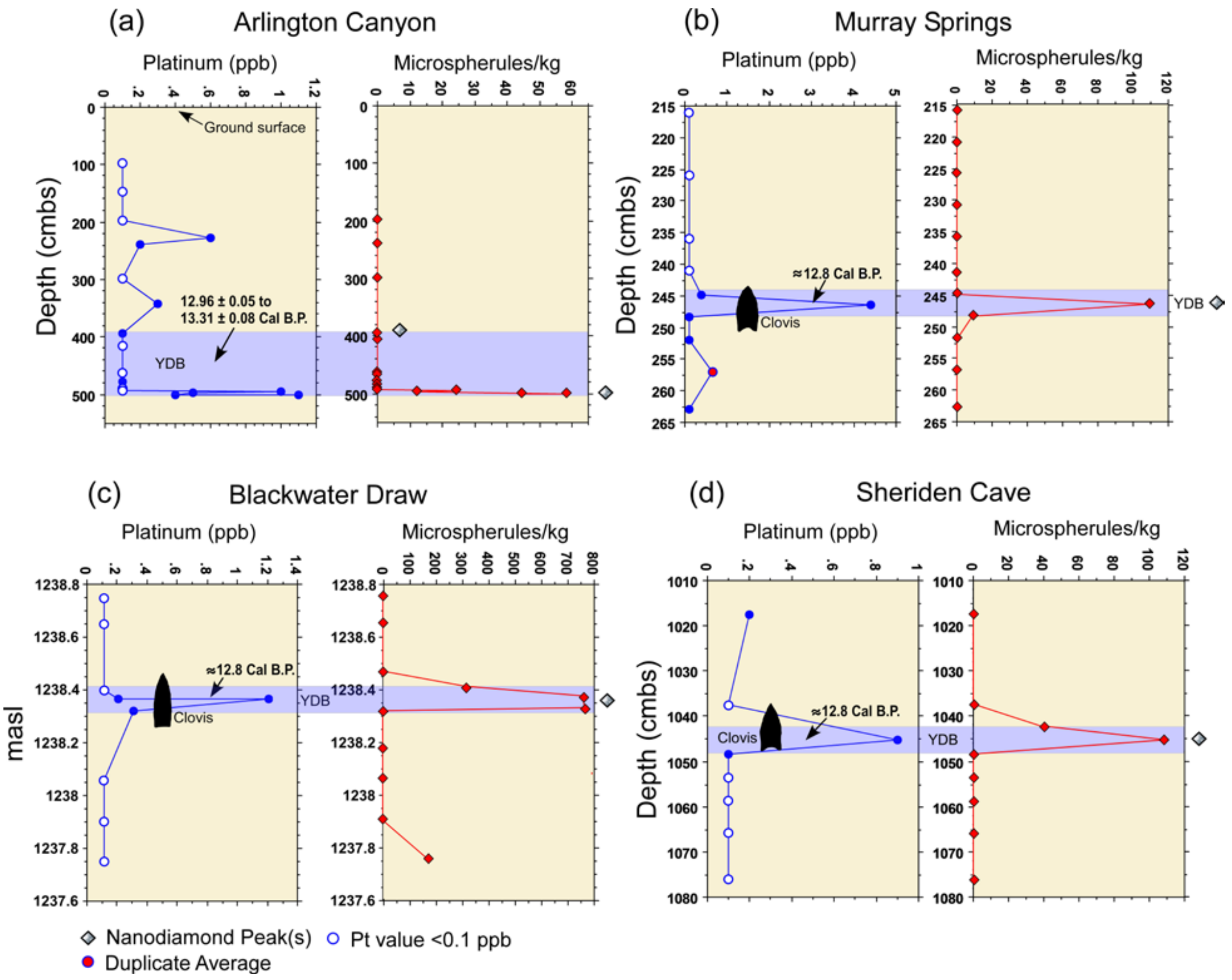

Supplementary Figure 2. Site graphs for western and Midwestern study sites (**a** to **d**). Graphs show platinum (Pt) abundance in ppb (error = +/- 0.1 ppb), archaeostratigraphic data (Paleoindian Clovis hafted biface silhouettes), microspherule abundance as microspherules per kilogram of sediment[2], the location of nanodiamond peaks[3-5,9], chronometric dates (radiocarbon [Cal B.P.]), and interpreted YDB. Each data value is plotted in the middle of the sample interval. The chronostratigraphic position of the YDB for each site was determined based on (**a**) linear interpolation of 12 AMS dates; (**b**) interpolation of 7 conventional and AMS radiocarbon dates based on second-order polynomial regression; (**c**) logarithmic interpolation of 5 conventional and AMS radiocarbon dates; (**d**) 3 AMS dates selected from the YDB layer[2], and the stratigraphic position of temporally diagnostic hafted bifaces. A Bayesian analysis of dates from all western and Midwestern study sites demonstrates synchronous deposition of the YDB layer within the limits of dating uncertainty (~100 y)[72].

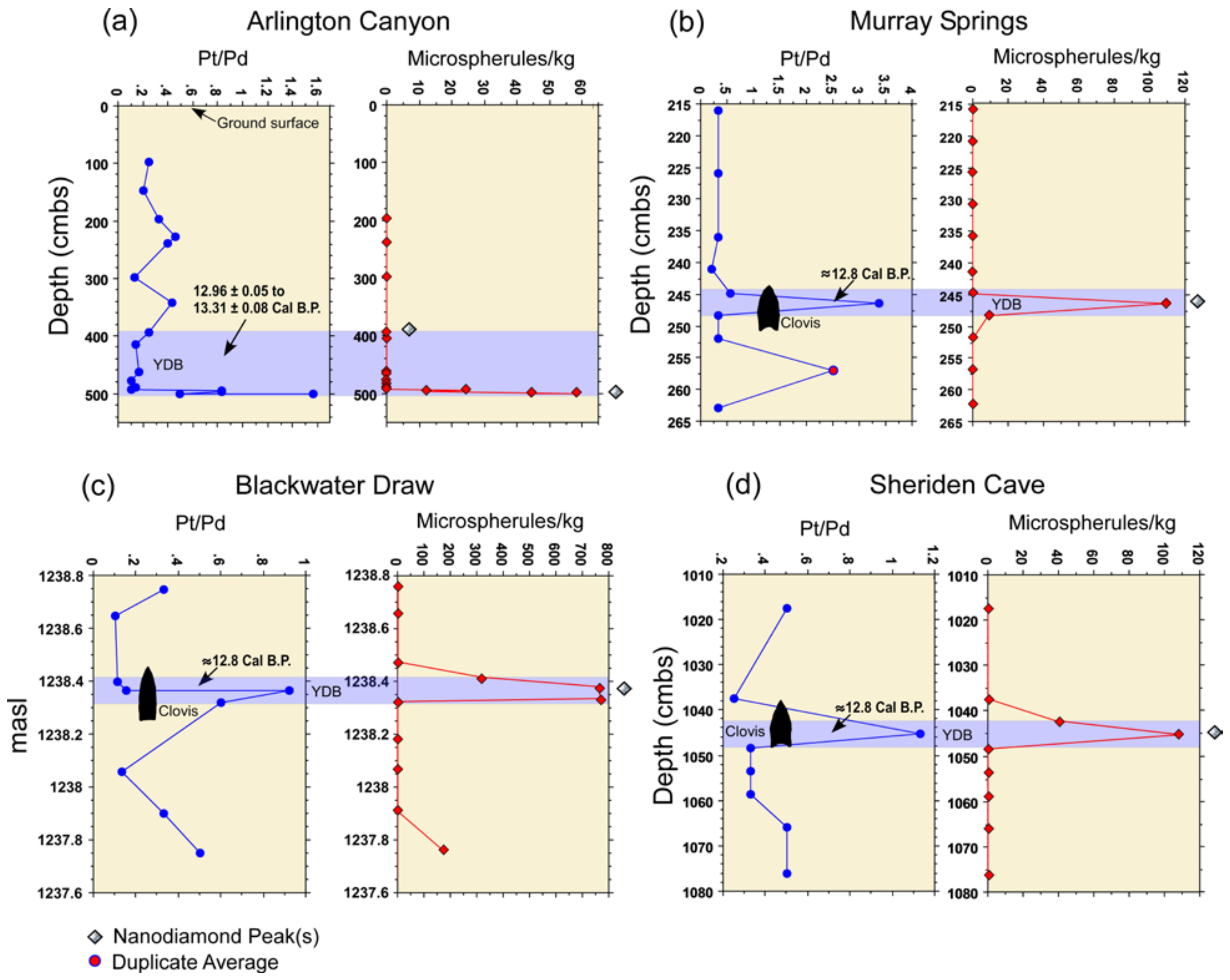

Supplementary Figure 3. Site graphs for western and Midwestern study sites (**a** to **d**). Graphs show the ratio of platinum to palladium (Pt/Pd), microspherule abundance as microspherules per kilogram of sediment[2], archaeostratigraphic data (Paleoindian Clovis hafted biface silhouettes), the location of nanodiamond peaks[3-5,9], chronometric dates (radiocarbon [Cal B.P.]), and interpreted YDB. Each data value is plotted in the middle of the sample interval. The chronostratigraphic position of the YDB for each site was determined based on (**a**) linear interpolation of 12 AMS dates; (**b**) interpolation of 7 conventional and AMS radiocarbon dates based on second-order polynomial regression; (**c**) logarithmic interpolation of 5 conventional and AMS radiocarbon dates, (**d**) 3 AMS dates selected from the YDB layer[2], and the stratigraphic position of temporally diagnostic hafted bifaces. A Bayesian analysis of dates from all western and Midwestern study sites demonstrates synchronous deposition of the YDB layer within the limits of dating uncertainty (~100 y)[72].

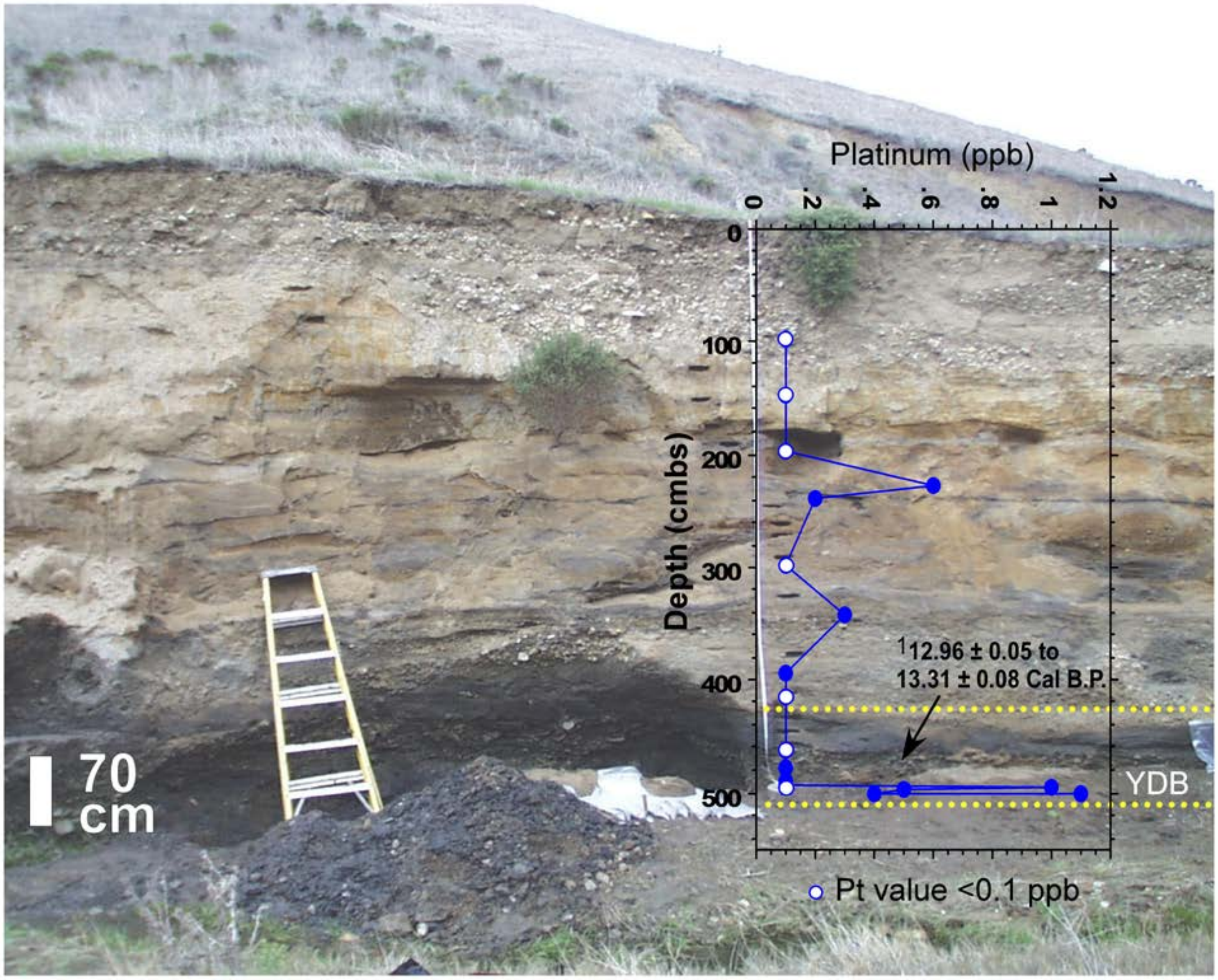

Supplementary Figure 4. Platinum (Pt) abundance (error = +/- 0.1 ppb) shown over the 5-meter-high cliff profile at Arlington Canyon (Site AC-003). The YDB layer is indicated between the yellow dotted lines. Figure is modified from Wittke *et al.*[2].

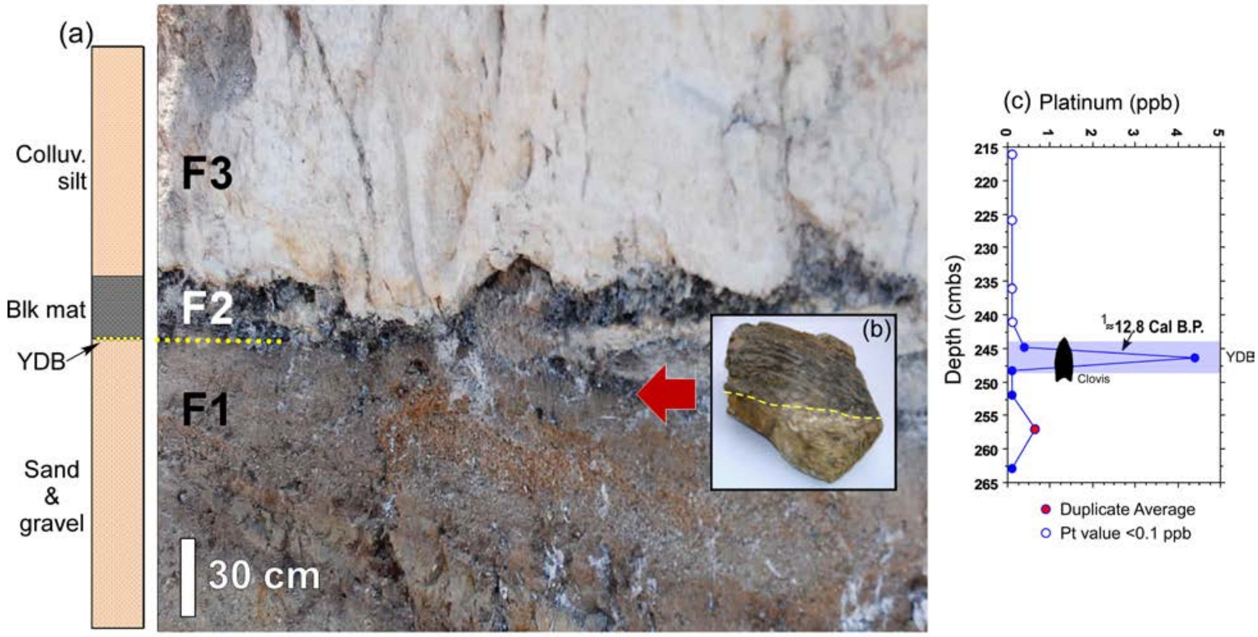

Supplementary Figure 5. Profile for Murray Springs showing (**a**) lithostratigraphic data, the location of the black mat (dark layer) and YDB layer (yellow dotted line), (**b**) an *in-situ* black mat stained mammoth tooth with its location indicated by a red arrow, and (**c**) platinum (Pt) abundance (error = +/- 0.1 ppb) along with a representation of a Clovis point found nearby in the YDB layer[8]. Figure is modified from Wittke *et al.*[2].

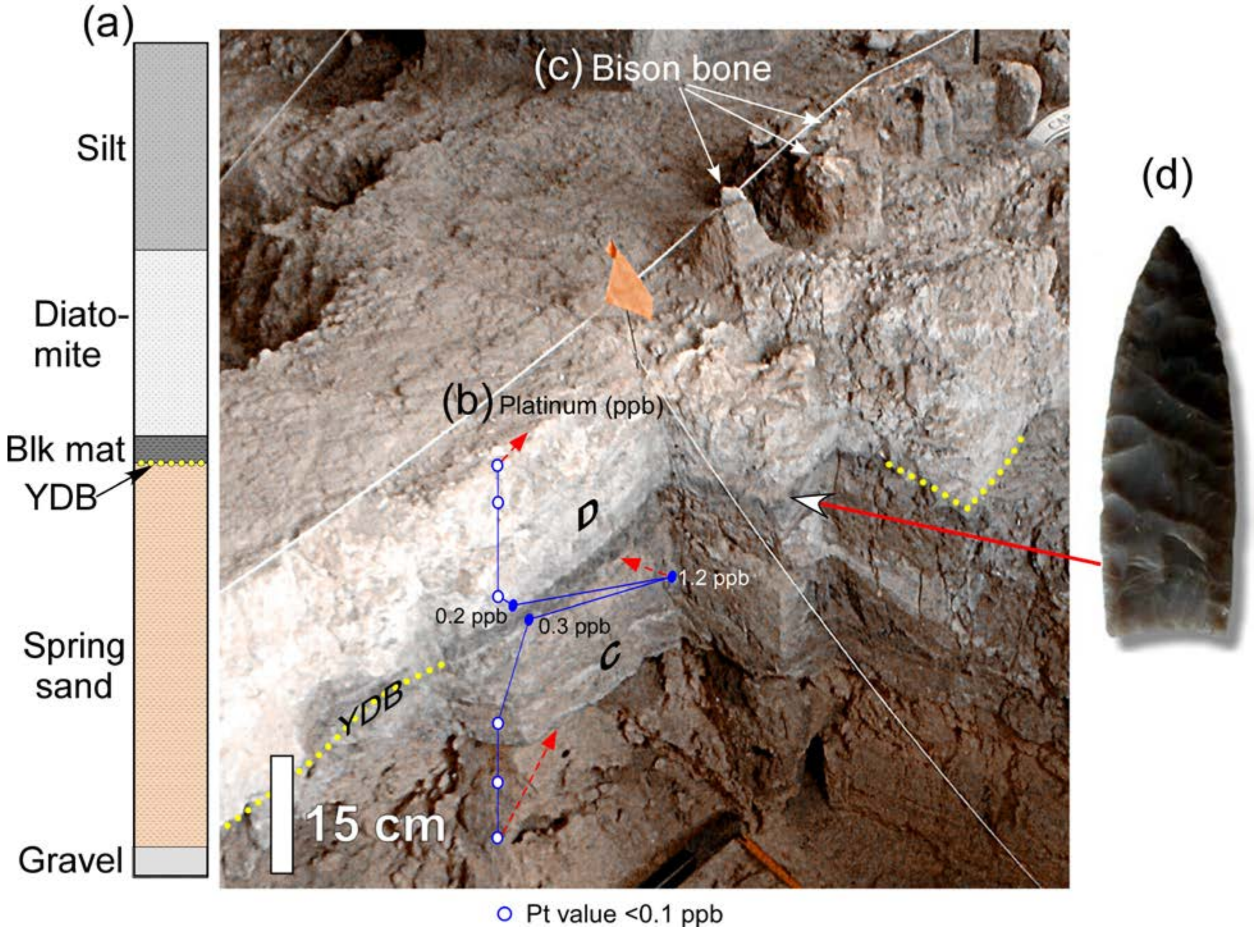

Supplementary Figure 6. Profile for Blackwater Draw showing (**a**) lithostratigraphic data, the location of the black mat (dark layer) and YDB layer (yellow dotted line), (**b**) platinum (Pt) abundance (error = +/- 0.1 ppb) plotted over the sampled profile, (**c**) bison bones from a post-Clovis Folsom-age occupation, and (**d**) a Clovis point found nearby in the YDB layer at the equivalent depth of red arrow. Figure is modified from Wittke *et al.*[2].

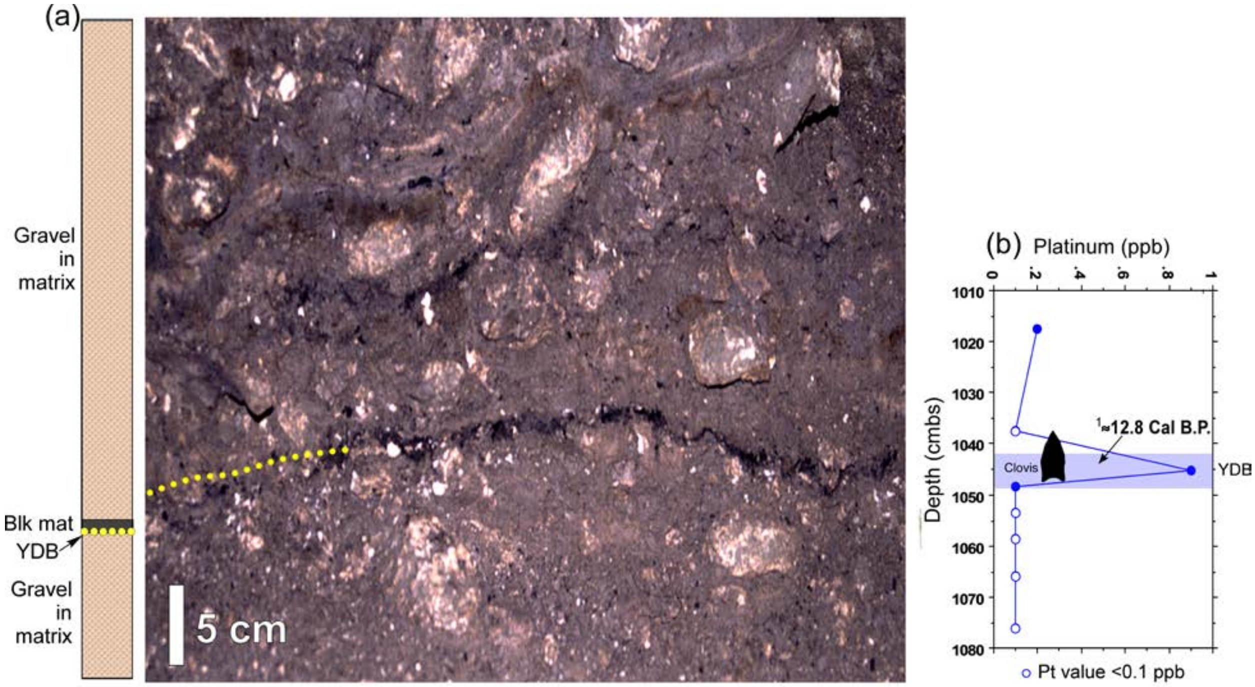

Supplementary Figure 7. A portion of the excavation profile for Sheriden Cave showing (**a**) lithostratigraphic data, the location of the black mat (dark layer) and YDB layer (yellow dotted line), and (**b**) platinum (Pt) abundance (error = +/- 0.1 ppb) along with a representation of a Clovis point found in the YDB layer. Figure is modified from Wittke *et al.*[2].

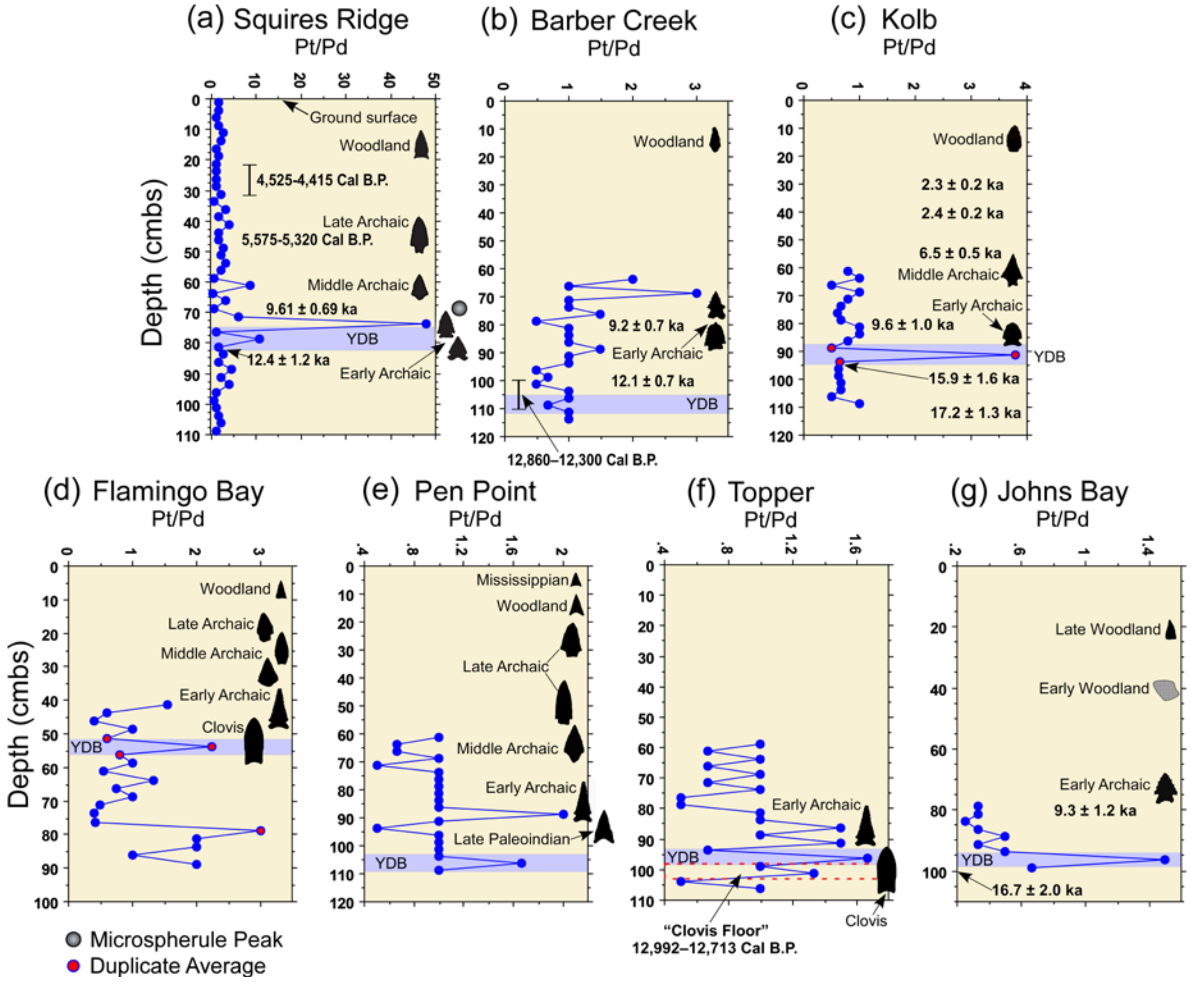

Supplementary Figure 8. Site graphs for eastern sites (**a** to **g**). Graphs show the ratio of platinum to palladium (Pt/Pd), generalized archaeostratigraphic data (Paleoindian through Woodland hafted biface silhouettes), chronometric dates (OSL [ka] and radiocarbon [Cal B.P.]), depth of microspherule peak (Squires Ridge only), and interpreted YDB. Each sample is plotted in the middle of the sample interval. (**b**) Radiocarbon date from Level 11 (100-110 cmbs) at Barber Creek is from an adjacent excavation unit. (**f**) Radiocarbon date from Clovis occupation surface "Clovis Floor" at Topper is from an adjacent excavation block. In a paper by Kennett *et al.*[72], a Bayesian analysis of dates from Topper and Barber Creek demonstrated synchronous deposition of the YDB layer within the limits of dating uncertainty (~100 y). See Supplementary Figures 9-15 and Supplementary Tables 4-6 for more detail on stratigraphy and dating.

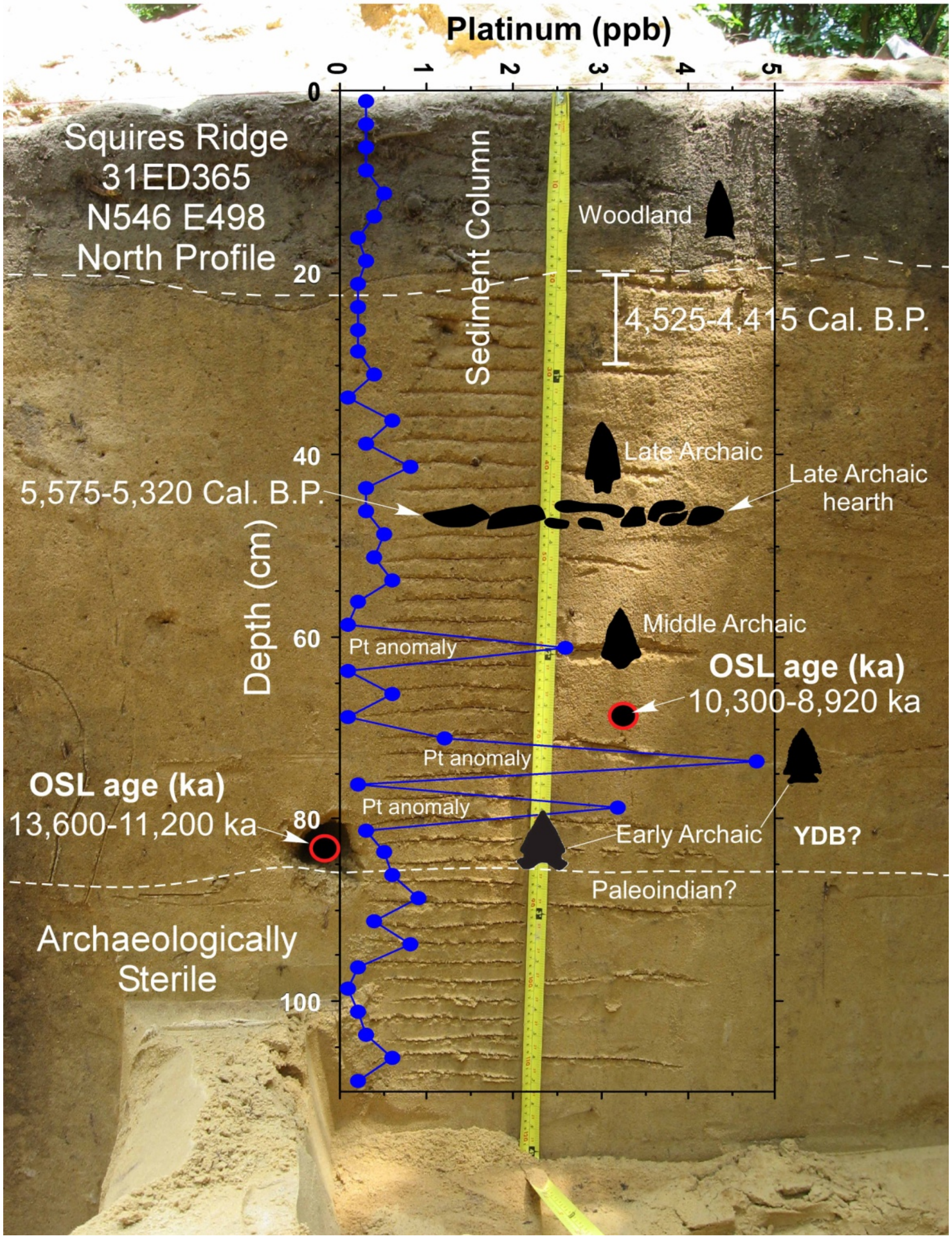

Supplementary Figure 9. Platinum (Pt) abundance (error = +/- 0.1 ppb) shown over the sampled excavation profile at Squires Ridge (31ED365), along with single-grain OSL and AMS dates (Supplementary Tables 5 and 6) and archaeostratigraphic data. Hafted bifaces represented by silhouettes reflect a generalized archaeostratigraphy over the entire excavation trench at Squires Ridge.

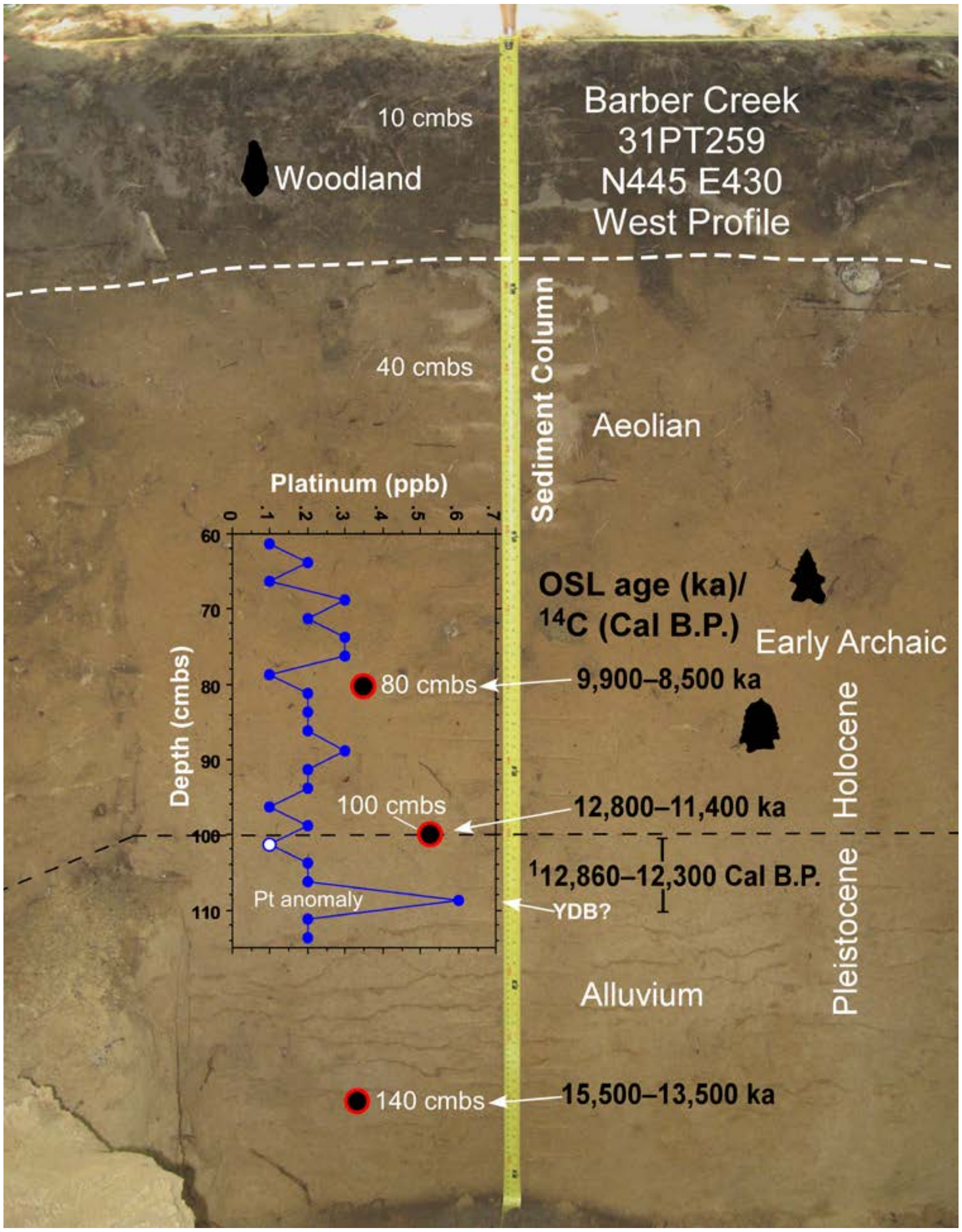

Supplementry Figure 10. Platinum (Pt) abundance (error = +/- 0.1 ppb) shown over the sampled excavation profile at Barber Creek (31PT259), along with single-grain OSL and AMS dates (Supplementary Tables 5 and 6), and the approximate location of the Pliestocene-Holocene boundary. Hafted bifaces represented by silhouettes reflect a generalized archaeostratigraphy for this unit and adjacent excavation units at Barber Creek. [1]The radiocarbon date is from an adjacent excavation block from Level 11 (100-110 cmbs). The white circle represents a Pt value measured as < 0.1 ppb.

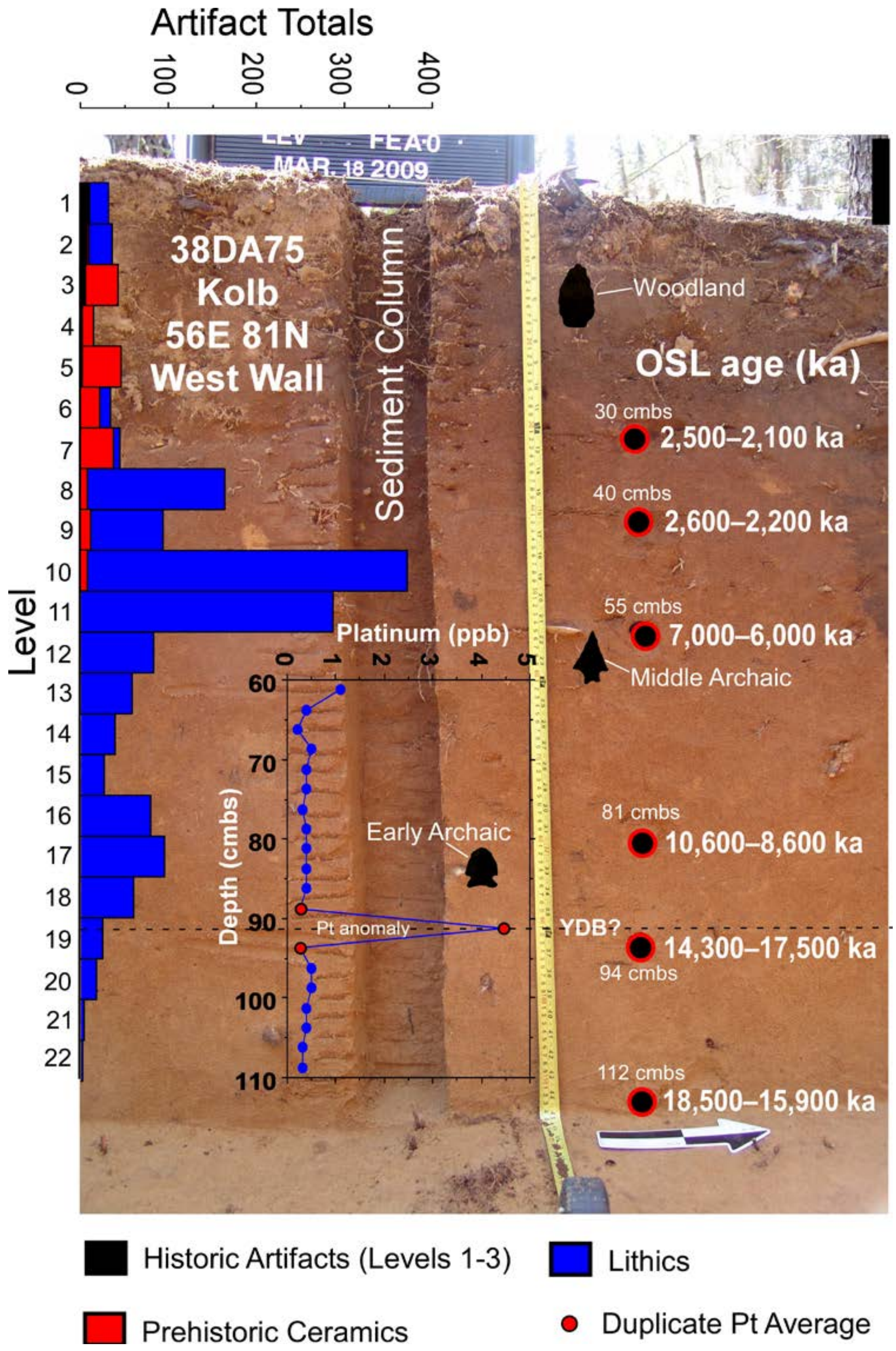

Supplementary Figure 11. Platinum (Pt) abundance (error = +/- 0.1 ppb) shown over the sampled excavation profile at Kolb (38DA75), along with single-grain OSL [ka] age estimates (Supplementary Table 6) and archaeostratigraphic data. Hafted bifaces silhouettes are shown at their measured depth below surface from the sampled excavation unit.

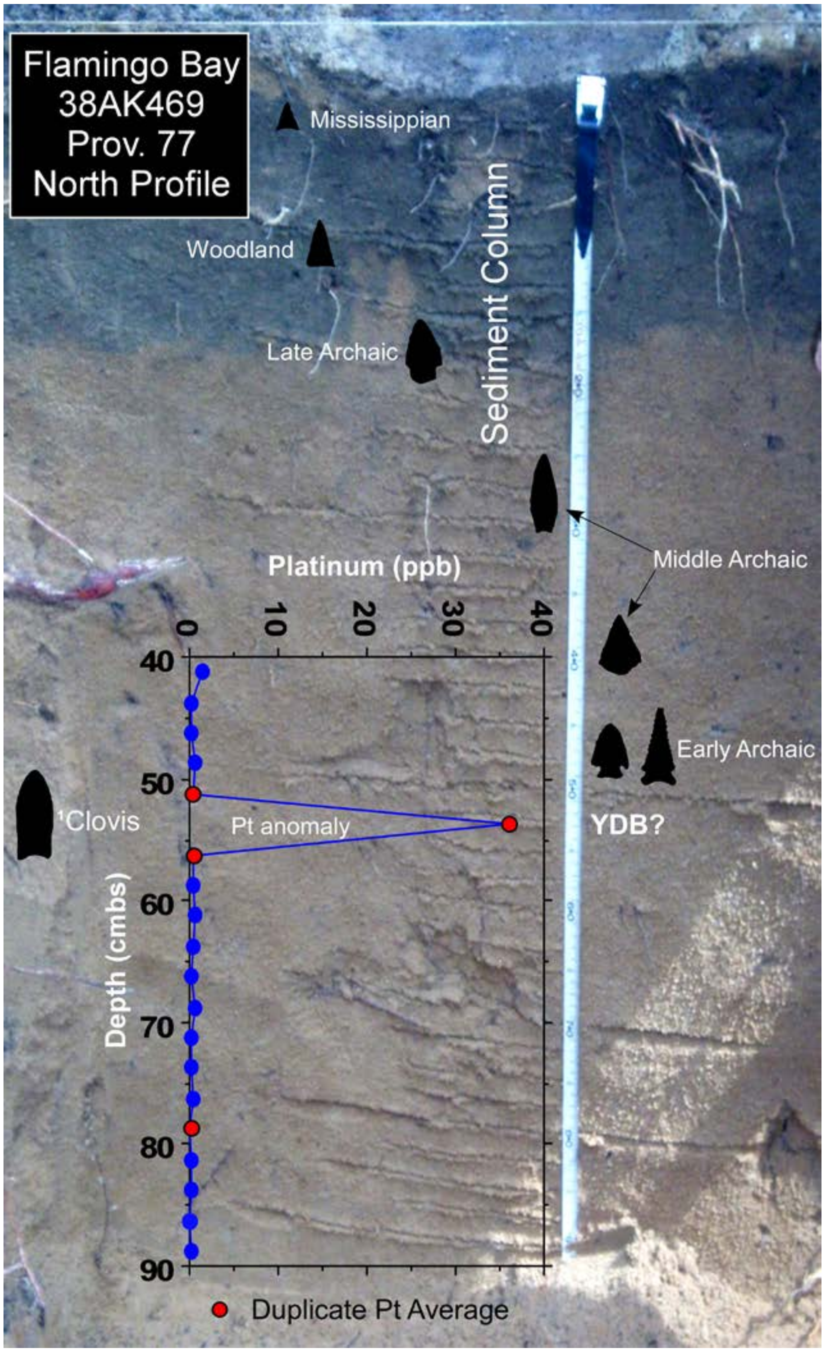

Supplementary Figure 12. Platinum (Pt) abundance (error = +/- 0.1 ppb) shown over the sampled excavation profile at Flamingo Bay (38AK469), along with the stratigraphic position of temporally diagnostic hafted bifaces. Hafted bifaces represented by silhouettes reflect a generalized archaeostratigraphy for the downslope portion of the main excavation block at Flamingo Bay. [1]The accepted date range for Clovis is ca. 13,250-12,850 Cal. B.P.[41] and overlaps with the lower boundary of the Younger Dryas Chronozone estimated at ca. 50-55 cmbs.

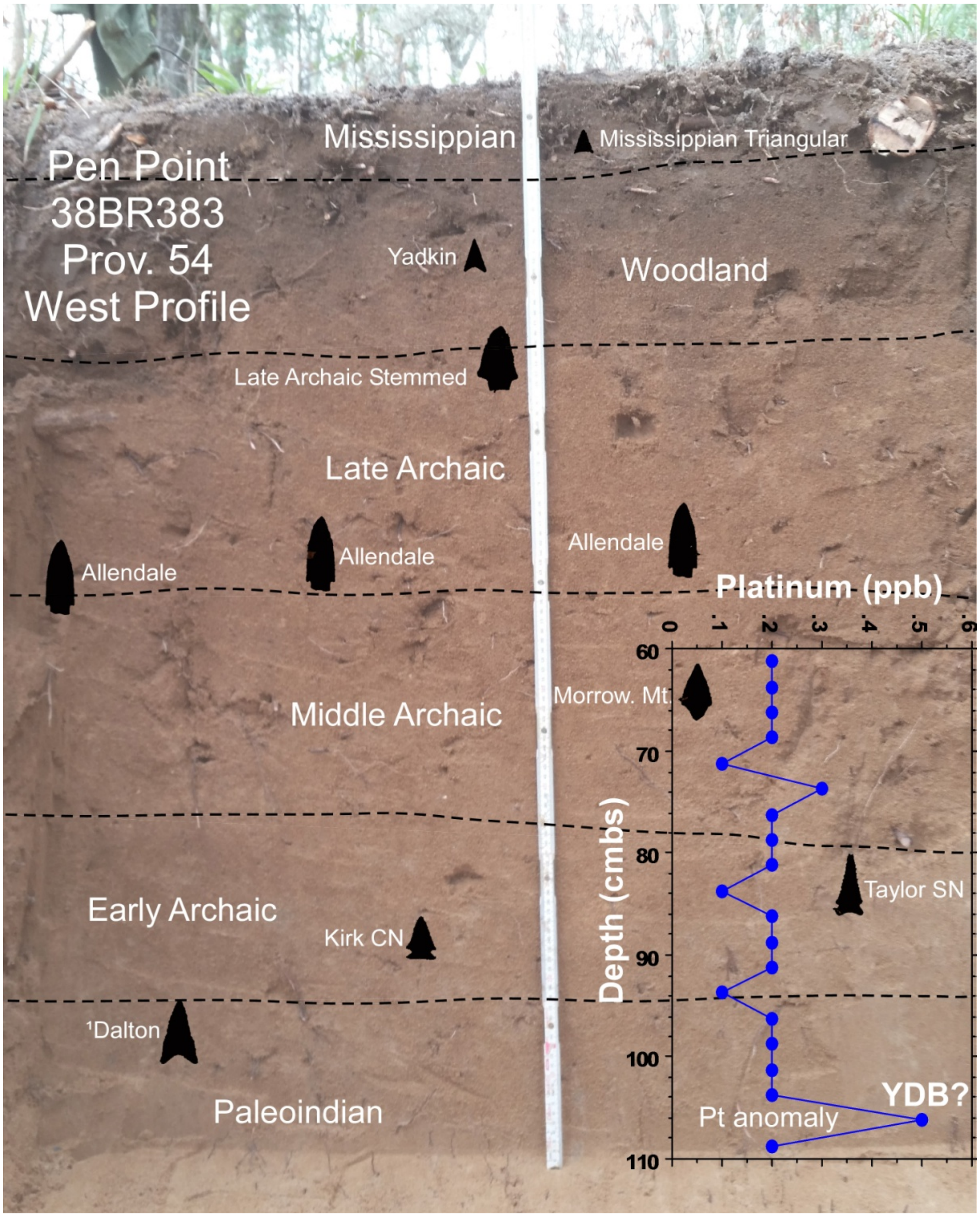

Supplementary Figure 13. Platinum (Pt) abundance (error = +/- 0.1 ppb) shown over the sampled excavation profile at Pen Point (38BR383), along with interpreted sedimentological and archaeostraigraphic zones, and the stratigraphic position of temporally diagnostic hafted bifaces. Hafted bifaces are shown based on the their measured depth and position in this excavation unit and those within the same excavation block. [1]The accepted date range for Dalton is ca. 12,500-11,300 Cal. B.P.[43] during the middle to later part of the Younger Dryas chronozone.

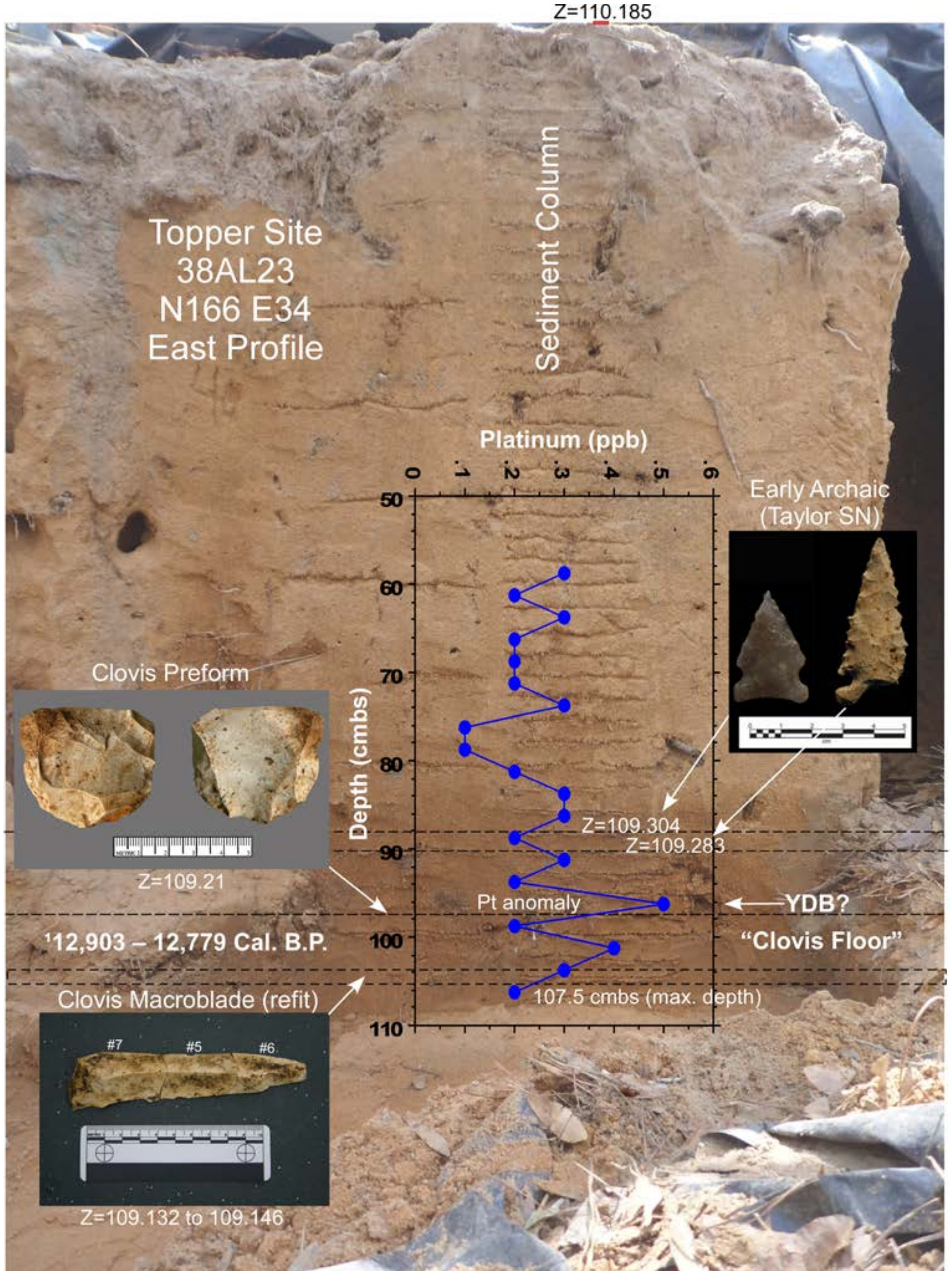

Supplementry Figure 14. Platinum (Pt) abundance (error = +/- 0.1 ppb) shown over the sampled excavation profile at Topper (38AL23), along with the stratigraphic position of temporally diagnostic Early Archaic and Clovis-age artifacts found in the sampled excavation and an [1]AMS date (Supplementary Table 5) of the Clovis occupation[44-50]. Elevation values (Z) are shown for artifacts and sediment column data. The "Clovis Floor" is a distinctive occupation surface identifiable across much of the site by the presence of dense quarry debris and Clovis tools[49]. The accepted date range for Clovis is ca. 13,250-12,850 Cal. B.P.[41].

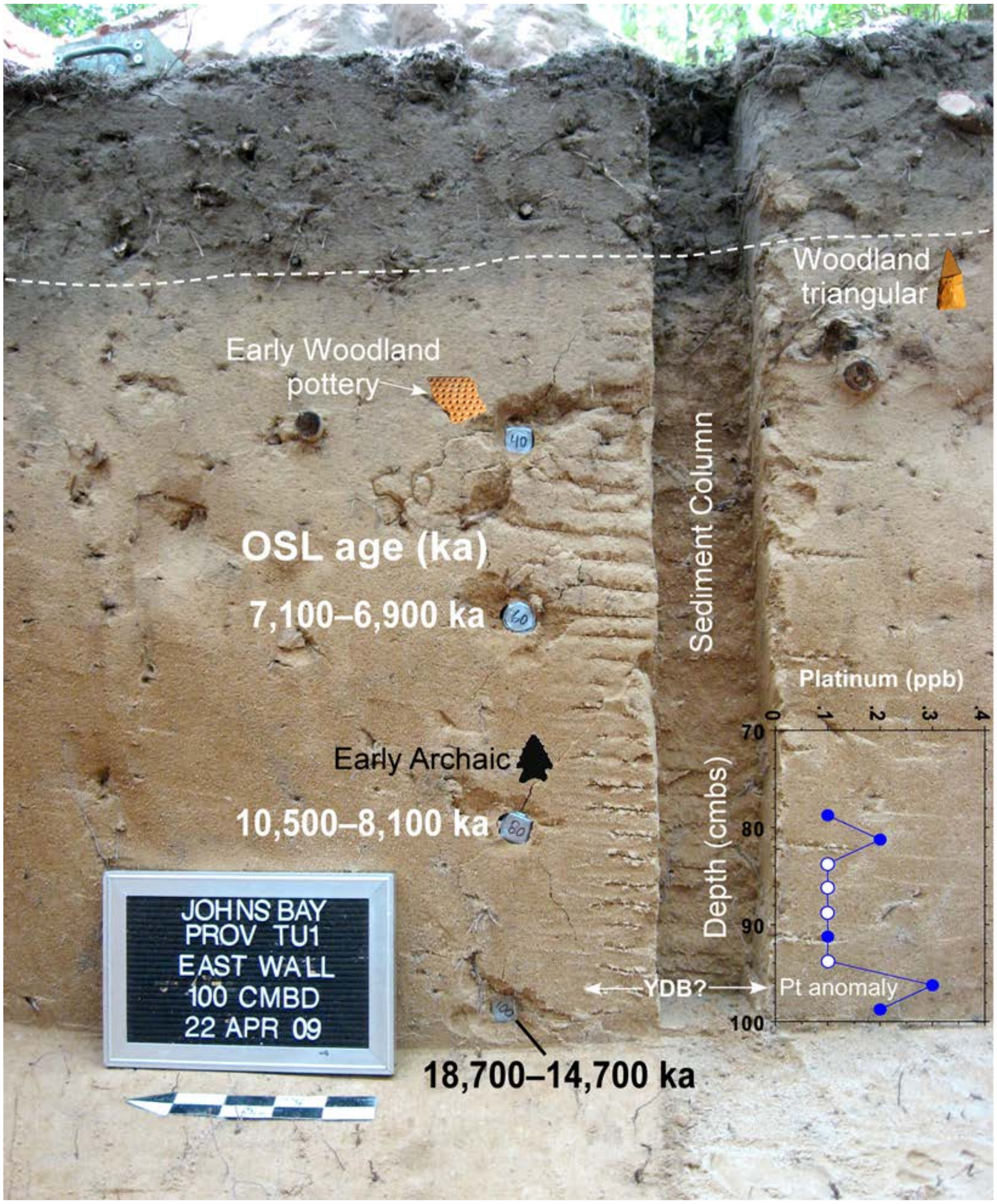

Supplementary Figure 15. Platinum (Pt) abundance (error = +/- 0.1 ppb) shown over the sampled excavation profile at Johns Bay (38AL246), along with with single-grain OSL [ka] age estimates (Supplementary Table 6), and the stratigraphic position of temporally diagnostic artifacts found in this excavation unit. White circles are Pt values that were measured as < 0.1 ppb.

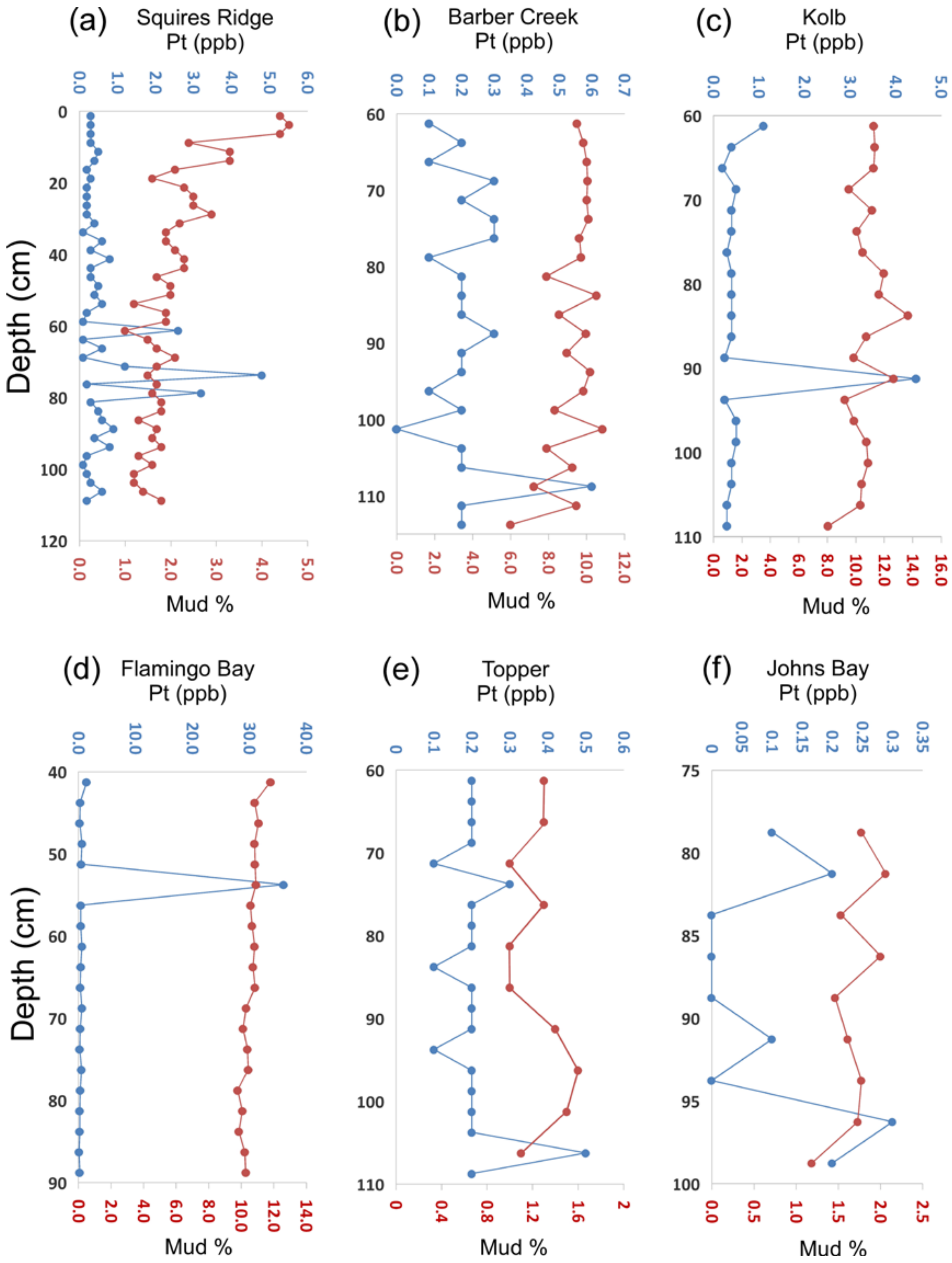

Supplementary Figure 16. Platinum (Pt) abundance (error = +/- 0.1 ppb) compared with percent mud (silt+clay) calculated for six sites (a to f). See Supplementary Table 9.

Supplementary Information Table 1. Pt, Pd, and Pt/Pd data for eastern study sites (n = 7).

Site Name	Squires Ridge			Barber Creek			Kolb			Flamingo Bay			Pen Point			Topper			Johns Bay		
Site #	31ED365			31PT259			38DA75			38AK469			38BR383			238AL23			38AL246		
Analyte Symbol	Pt	Pd	Pt/Pd	Pt	Pd	Pt/Pd	Pt	Pd	Pt/Pd	Pt	Pd	Pt/Pd	Pt	Pd	Pt/Pd	Pt	Pd	Pt/Pd	Pt	Pd	Pt/Pd
Units	ppb			ppb			ppb			ppb			ppb			ppb			ppb		
Detection Limit	0.1			0.1			0.1			0.1			0.1			0.1			0.1		
Analysis Method	FA-MS			FA-MS			FA-MS			FA-MS			FA-MS			FA-MS			FA-MS		
/[1]Depth (cm)																					
1.25	0.3	0.2	1.5																		
3.75	0.3	0.2	1.5																		
6.25	0.3	0.3	1.0																		
8.75	0.3	0.2	1.5																		
11.25	0.5	0.2	2.5																		
13.75	0.4	0.2	2.0																		
16.25	0.2	0.2	1.0																		
18.75	0.3	0.2	1.5																		
21.25	0.2	0.2	1.0																		
23.75	0.2	0.2	1.0																		
26.25	0.2	0.2	1.0																		
28.75	0.2	0.2	1.0																		
31.25	0.4	0.2	2.0																		
33.75	0.1	0.2	0.5																		
36.25	0.6	0.2	3.0																		
38.75	0.3	0.2	1.5																		
41.25	0.8	0.2	4.0							1.4	0.9	1.6									
43.75	0.3	0.2	1.5							0.3	0.5	0.6									
46.25	0.3	0.2	1.5							0.2	0.5	0.4									
48.75	0.5	0.2	2.5							0.6	0.6	1.0									
51.25	0.4	0.2	2.0							0.5	0.6	0.8									
51.25 (dup)										0.4	1	0.4									
53.75	0.6	0.2	3.0							**65.6**	**133**	**0.5**									
53.75 (dup)										**6.4**	**1.6**	**4.0**									
56.25	0.2	0.1	2.0							0.3	0.5	0.6									
56.25 (dup)										0.5	0.5	1.0									
58.75	0.1	0.2	0.5							0.4	0.4	1.0				0.3	0.3	1.0			
61.25	**2.6**	**0.3**	**8.7**	0.1	< 0.1	1.0	1.1	1.4	0.8	0.6	1.1	0.5	0.2	0.2	1.0	0.2	0.3	0.7			
63.75	0.1	0.3	0.3	0.2	0.1	2.0	0.4	0.4	1.0	0.4	0.3	1.3	0.2	0.3	0.7	0.3	0.3	1.0			
66.25	0.6	0.2	3.0	0.1	0.1	1.0	0.2	0.4	0.5	0.3	0.4	0.8	0.2	0.3	0.7	0.2	0.3	0.7			
68.75	0.1	0.2	0.5	0.3	0.1	3.0	0.5	0.5	1.0	0.6	0.6	1.0	0.2	0.2	1.0	0.2	0.2	1.0			
71.25	**1.2**	**0.2**	**6.0**	0.2	0.2	1.0	0.4	0.5	0.8	0.3	0.6	0.5	0.1	0.2	0.5	0.2	0.3	0.7			
73.75	**4.8**	**0.1**	**48.0**	0.3	0.3	1.0	0.4	0.6	0.7	0.2	0.5	0.4	0.3	0.3	1.0	0.3	0.3	1.0			
76.25	0.2	0.2	1.0	0.3	0.2	1.5	0.3	0.5	0.6	0.5	1.2	0.4	0.2	0.2	1.0	0.1	0.2	0.5			
78.75	**3.2**	**0.3**	**10.7**	0.1	0.2	0.5	0.4	0.6	0.7	0.5	0.1	5.0	0.2	0.2	1.0	0.1	0.2	0.5	0.1	0.3	0.3
78.75 (dup)										<0.1	0.1	1.0									
81.25	0.3	0.2	1.5	0.2	0.2	1.0	0.4	0.4	1.0	0.2	0.1	2.0	0.2	0.2	1.0	0.2	0.2	1.0	0.2	0.6	0.3
83.75	0.5	0.2	2.5	0.2	0.2	1.0	0.4	0.4	1.0	0.2	0.1	2.0	0.1	0.1	1.0	0.3	0.3	1.0	< 0.1	0.4	0.3
86.25	0.6	0.4	1.5	0.2	0.2	1.0	0.4	0.5	0.8	0.1	0.1	1.0	0.2	0.2	1.0	0.3	0.2	1.5	< 0.1	0.3	0.3
88.75	0.9	0.2	4.5	0.3	0.2	1.5	0.4	0.6	0.7	0.2	0.1	2.0	0.2	0.1	2.0	0.2	0.2	1.0	< 0.1	0.2	0.3
88.75 (dup)							<0.1	0.4	0.3												
91.25	0.4	0.2	2.0	0.2	0.2	1.0	**8.4**	**1.2**	**7.0**				0.2	0.2	1.0	0.3	0.2	1.5	0.1	0.3	0.3
91.25 (dup)							**0.5**	**0.9**	**0.6**												
93.75	0.8	0.2	4.0	0.2	0.2	1.0	0.4	0.4	1.0				0.1	0.2	0.5	0.2	0.3	0.7	< 0.1	0.2	0.5
93.75 (dup)							<0.1	0.3	0.3												
96.25	0.2	0.2	1.0	0.1	0.2	0.5	0.5	0.8	0.6				0.2	0.2	1.0	**0.5**	**0.3**	**1.7**	**0.3**	**0.2**	**1.5**
98.75	0.1	0.2	0.5	0.2	0.3	0.7	0.5	0.8	0.6				0.2	0.2	1.0	0.2	0.2	1.0	0.2	0.3	0.7
101.25	0.2	0.2	1.0	< 0.1	0.2	0.5	0.4	0.6	0.7				0.2	0.2	1.0	0.4	0.3	1.3			
103.75	0.3	0.2	1.5	0.2	0.2	1.0	0.4	0.6	0.7				0.2	0.2	1.0	0.3	0.6	0.5			
106.25	0.6	0.3	2.0	0.2	0.2	1.0	0.3	0.6	0.5				**0.5**	**0.3**	**1.7**	0.2	0.2	1.0			
108.75	0.2	0.2	1.0	**0.6**	**0.9**	**0.7**	0.3	0.3	1.0				0.2	0.2	1.0						
111.25				0.2	0.2	1.0															
113.75				0.2	0.2	1.0															

[1]Depths are midpoints for sample increments.

Note: Pt anomaly samples are shown in red.

Supplementary Information Table 2. Pt, Pd, and Pt/Pd data for western and Midwestern study sites (n = 4).

Site Name	Arlington Canyon			Murray Springs			Blackwater Draw			Sheriden Cave		
Analyte Symbol	Pt	Pd	Pt/Pd	Pt	Pd	Pt/Pd	Pt	Pd	Pt/Pd	Pt	Pd	Pt/Pd
Unit Symbol		ppb			ppb			ppb			ppb	
Detection Limit		0.1			0.1			0.1			0.1	
Analysis Method		FA-MS			FA-MS			FA-MS			FA-MS	
[1]Depth (cm) /Elev (masl)												
216				<0.1	0.3	0.3						
226				<0.1	0.3	0.3						
236				<0.1	0.3	0.3						
241				<0.1	0.5	0.2						
244.8				0.4	0.7	0.6						
246.5				**4.4**	**1.3**	**3.4**						
248.3				0.1	0.3	0.3						
252				0.1	0.3	0.3						
257				1.2	0.3	4.0						
257 (dup)				<0.1	0.1	1.0						
263				0.1	0.3	0.3						
1238.75 masl							< 0.1	0.3	0.3			
1238.65 masl							< 0.1	1	0.1			
1238.4 masl							< 0.1	0.9	0.1			
1238.365 masl							0.2	1.3	0.2			
1238.365 masl							**1.2**	**1.3**	**0.9**			
1238.32 masl							0.3	0.5	0.6			
1238.06 masl							< 0.1	0.8	0.1			
1237.9 masl							< 0.1	0.3	0.3			
1237.75 masl							< 0.1	0.2	0.5			
97.0	< 0.1	0.4	0.25									
146.5	< 0.1	0.5	0.20									
196.5	< 0.1	0.3	0.33									
227.5	0.6	1.3	0.46									
239.5	0.2	0.5	0.40									
298.5	< 0.1	0.8	0.13									
341.5	0.3	0.7	0.43									
394.0	0.1	0.4	0.25									
414.5	< 0.1	0.7	0.14									
461.5	< 0.1	0.6	0.17									
477.5	0.1	0.9	0.11									
488.0	< 0.1	0.7	0.14									
492.0	< 0.1	0.9	0.11									
495.5	**1**	**1.2**	**0.83**									
497.0	**0.5**	**0.6**	**0.83**									
500.5	**0.4**	**0.8**	**0.50**									
501.0	**1.1**	**0.7**	**1.57**									
1017.5										0.2	0.4	0.50
1037.5										<0.1	0.4	0.25
1045.3										**0.9**	**0.8**	**1.13**
1048.5										0.1	0.3	0.33
1053.5										<0.1	0.3	0.33
1058.5										<0.1	0.3	0.33
1066										<0.1	0.2	0.50
1076										<0.1	0.2	0.50

[1]Depths are midpoints for sample increments; masl = meters above sea-level.
Note: Pt anomaly samples are shown in red.

Supplementary Information Table 3. Attributes of western and Midwestern study sites (n = 4).

Site Name	Location		Landform Type	Depositional Environment	Earliest Cultural Component	Chronology	Sample Strategy/ Increment
	Latitude	Longitude					
Arlington Canyon	33.990333°N	120.158056°W	Stream-Cut Cliff	Alluvial	Early Paleoindian	^{14}C	Discontinuous/Variable
Murray Springs	31.570912°N	110.177996°W	Alluvial Terrace	Alluvial/Colluvial/ Marl/Lacustrine	Early Paleoindian	^{14}C	Discontinuous/Variable
Blackwater Draw	34.275687°N	103.326101°W	Alluvial Terrace	Alluvial/Lacustrine	Early Paleoindian	^{14}C	Discontinuous/Variable
Sheriden Cave	40.965055°N	83.426038°W	Cave	Freeze-thaw Cycles and Solifluction	Early Paleoindian	^{14}C	Continuous/Variable

Supplementary Information Table 4. Attributes of eastern study sites (n = 7).

Site Name	Site #	Location		Landform Type	Depositional Environment	Earliest Cultural Component	Chronology	Sample Strategy/Increment
		Latitude	Longitude					
Squires Ridge	31ED365	35.841218°	77.541847°	Alluvial Terrace	Aeolian/Fluvial Overbank	Early Archaic	Archaeostratigraphy, OSL, and ^{14}C	Continuous/2.5 cm
Barber Creek	31PT259	35.600882°	77.304065°	Alluvial Terrace	Aeolian/Fluvial Overbank	Early Archaic	Archaeostratigraphy, OSL, and ^{14}C	Continuous/2.5 cm
Kolb	38DA75	34.381765°	79.711102°	Alluvial Terrace	Fluvial Overbank	Early Paleoindian	Archaeostratigraphy and OSL	Continuous/2.5 cm
Flamingo Bay	38AK469	33.337398°	81.677552°	Carolina bay sand rim	Lacustrine/Aeolian	Early Paleoindian	Archaeostratigraphy and OSL	Continuous/2.5 cm
Pen Point	38BR383	33.142181°	81.699025°	Alluvial Terrace	Fluvial Overbank	Late Paleoindian	Archaeostratigraphy	Continuous/2.5 cm
Topper	38AL23	33.005439°	81.490424°	Hillslope	Colluvial/Slopewash	Early Paleoindian	Archaeostratigraphy and ^{14}C	Continuous/2.5 cm
Johns Bay	38AL246	33.017649°	81.273274°	Carolina bay sand rim	Lacustrine/Aeolian	Early Archaic	Archaeostratigraphy and OSL	Continuous/2.5 cm

Table S5. Radiocarbon dates for eastern study sites.

Site Name	Site #	Method	Radiocarbon Age	[1]Cal BP	Beta Number
Squires Ridge	31ED365	AMS	3990 ± 30	4525–4415	Beta-414621
Squires Ridge	31ED365	AMS	4690 ± 30	5575–5540; 5475–5435; 5425–5420	Beta-414622
Barber Creek	31PT259	AMS	10,500 ± 50	12,860–12,300	Beta-188956
Topper	38AL23	AMS	10,958 ± 65	12,992–12,713	AA-100294

[1]*INTCAL04 (2 Sigma) calibration.*

Table S6. OSL dosimetry data and basis for age for eastern sites.

[1] Sample	Site	[2] Method	Depth (cm)	^{238}U (ppm)	^{233}Th (ppm)	K (%)	Total dose rate (Gy/ka)	Central age D_e (Gy)	σ_b (%)	Minimum age D_e (Gy)	FMM–most common component (Gy)	Age (ka)	% error	[3] Basis for age
UW3134	Squires Ridge	s-g	67.5	1.11±0.09	1.90±0.53	1.27±0.03	1.77±0.07	17.0±0.8	38±4	11.1±1.0	17.5±0.9	9.6±0.6	6.2	CAM
UW3263	Squires Ridge	s-g	83	1.03±0.09	2.27±0.56	1.19±0.03	1.69±0.07	19.6±1.4	58±6	10.8±1.0	25.3±1.3	11.6±1.0	8.6	CAM
UW1907	Barber Creek	s-g	80	1.64±0.13	5.22±0.91	1.39±0.03	2.01±0.08	18.5±0.9	44±4	-	16.7±1.2	9.2±0.7	7.6	CAM
UW1908	Barber Creek	s-g	100	1.53±0.12	3.74±0.76	1.28±0.04	1.91±0.08	23.1±0.8	30±3	-	21.1±2.0	12.1±0.7	5.8	CAM
UW2725	Kolb	s-g	30	0.77±0.07	1.56±0.48	0.89±0.01	1.31±0.06	4.2±0.5	76±10	-	3.0±0.2	2.3±0.2	8.4	LC
UW2726	Kolb	s-g	40	0.62±0.07	2.46±0.54	0.96±0.04	1.38±0.07	4.5±0.7	93±12	-	3.3±0.3	2.4±0.2	10.3	LC
UW2710	Kolb	s-g	55	1.04±0.08	1.47±0.43	0.76±0.02	1.22±0.06	8.1±0.8	61±8	-	7.9±0.5	6.5±0.5	8.3	LC
UW2711	Kolb	s-g	81	1.17±0.10	2.80±0.65	0.86±0.04	1.41±0.07	11.1±0.8	40±6	-	13.5±1.2	9.6±1.0	10.4	LC
UW2709	Kolb	s-g	94	0.73±0.06	1.59±0.44	0.82±0.03	1.37±0.11	18.1±1.5	7	-	21.8±1.3	15.9±1.6	10.1	LC
UW2708	Kolb	s-g	112	0.64±0.07	2.63±0.62	0.88±0.03	1.28±0.06	17.4±1.6	64±7	-	22.0±1.1	17.2±1.3	7.4	LC
UW2143	Johns Bay	s-g	80	0.78±0.07	2.65±0.57	0.06±0.01	0.60±0.05	11.6±0.6	51±4	5.6±0.5	16.3±0.6	9.3±1.2	12.9	MAM
UW2144	Johns Bay	s-g	100	0.60±0.08	3.96±0.69	0.04±0.01	0.61±0.05	15.6±0.6	36±4	10.2±0.8	19.9±1.2	16.7±2.0	12	MAM

[1]UW, University of Washington; James Feathers.

[2]Dating method; s-g=single-grain; s-a=single aliquot.

[3]Basis for age; CAM=Central Age Model, MAM=Minimum Age Model, LC=Largest Component. Moisture content was taken as 6±3%, typical for sandy sediments in temperate climates (Brady 1974).

U, Th, and K values determined by UW by alpha counting, beta counting, and flame photometry. U, Th, and K values are determined by gamma spectrometry or ICP-MS.

Table S7. Pt and Pt/Pd data for multiple sources ([1]GERM) (*S64*).

Type	Pt-Hi	Pt-Lo	Pt-avg	Pt/Pd-Hi	Pt/Pd-Lo
Meteorite, iron (n=48)	39300.0	2610.0	16077.0	21.5	0.7
Meteorite, ureilite (n=24)	38000.0	100.0	12837.9	18.1	0.8
Meteorite, achondrite (n=47)	6700.0	0.2	1283.2	2.5	0.8
Meteorite, chondrite (n=48)	5838.0	478.0	1198.1	1.8	1.3
Impactites, 2.55 Ga (n=18)	380.0	0.7	33.2	6.4	1.0
Impactites, 65 Ma (n=60)	81.1	0.6	18.3	3.3	0.7
Impactites, 145 Ma (n=8)	49.0	23.0	42.1	1.9	1.8
Rocks, ocean trench (n=31)	13690.0	6.0	1206.2	44.1	3.0
Rocks, mantle, [2]MORB (n=8)	9350.0	7.0	2072.0	1.1	0.2
Rocks, mantle, craton (n=438)	315.0	0.2	9.8	1.3	0.3
Rocks, mantle, ultramafic (n=12)	277.0	18.0	57.5	7.5	0.7
Rocks, magma (n=24)	11.1	1.3	5.3	1.5	0.8

[1]GERM= (Geochemical Earth Reference Model) Reservoir Database

[2]MORB= Mid-Ocean Ridge Basalt

Supplementary Information Table 8. Pt, Ir, and Os detected by Andronikov[61] in microspherules from Blackwater Draw.

Mag Spherules	Pt (ppb)	Times Crustal	Ir (ppb)	Times Crustal	Os (ppb)	Times Crustal
Crustal (ppb)		0.5		0.022		0.031
MMs#1	80.6	161	1.89	86	bdl	--
MMs#2	bdl	--	bdl	--	bdl	--
MMs#3	22.1	44	bdl	--	bdl	--
MMs#4	18.2	36	1.48	67	1	36
MMs#5	460	920	5.05	230	8	247
MMs#6	bdl	--	0.410	19	3	104
Chondrites*	1200	2400	424	19273	500	16129
Iron metes*	16000	32000	7500	340909	26	839

*Source: GERM Database at https://earthref.org/GERMRD/

Supplementary Information Table 9. Pt data compared with percent mud (silt+clay) for eastern study sites where grain size data are available (n = 6).

Site Name	Squires Ridge		Barber Creek		Kolb		Flamingo Bay		Pen Point		Johns Bay	
/Depth (cm)	Pt (ppb)	Mud%	Pt (ppb)	Mud%	[a]Pt (ppb)	Mud%	[a]Pt (ppb)	Mud%	Pt (ppb)	[b]Mud%	Pt (ppb)	Mud%
0-2.5	0.3	4.4										
2.5-5	0.3	4.6										
5-7.5	0.3	4.4										
7.5-10	0.3	2.4										
10-12.5	0.5	3.3										
12.5-15	0.4	3.3										
15.17.5	0.2	2.1										
17.5-20	0.3	1.6										
20-22.5	0.2	2.3										
22.5-25	0.2	2.5										
25-27.5	0.2	2.5										
27.5-30	0.2	2.9										
30-32.5	0.4	2.2										
32.5-35	0.1	1.9										
35-37.5	0.6	1.9										
37.5-40	0.3	2.1										
40-42.5	0.8	2.3					1.4	11.8				
42.5-45	0.3	2.3					0.3	10.8				
45-47.5	0.3	1.7					0.2	11.1				
47.5-50	0.5	2.0					0.6	10.8				
50-52.5	0.4	2.0					**0.5**	10.8				
52.5-55	0.6	1.2					**36.0**	10.9				
55-57.5	0.2	1.9					**0.4**	10.6				
57.5-60	0.1	1.9					0.4	10.7				
60-62.5	2.6	1.0	0.1	9.5	1.1	11.3	0.6	10.8	0.2	1.3		
62.5-65	0.1	1.5	0.2	9.8	0.4	11.3	0.4	10.7	0.2			
65-67.5	0.6	1.7	0.1	10.0	0.2	11.3	0.3	10.8	0.2	1.3		
67.5-70	0.1	2.1	0.3	10.1	0.5	9.5	0.6	10.3	0.2			
70-72.5	1.2	1.7	0.2	10.0	0.4	11.1	0.3	10.1	0.1	1		
72.5-75	4.8	1.5	0.3	10.1	0.4	10.1	0.2	10.4	0.3			
75-77.5	0.2	1.7	0.3	9.6	0.3	10.5	0.5	10.4	0.2	1.3		
77.5-80	3.2	1.6	0.1	9.7	0.4	12.0	**0.3**	9.8	0.2		0.1	1.8
80-82.5	0.3	1.8	0.2	7.9	0.4	11.6	0.2	10.1	0.2	1	0.2	2.1
82.5-85	0.5	1.8	0.2	10.5	0.4	13.7	0.2	9.9	0.1		< 0.1	1.5
85-87.5	0.6	1.3	0.2	8.6	0.4	10.7	0.1	10.2	0.2	1	< 0.1	2.0
87.5-90	0.9	1.7	0.3	10.0	**0.3**	9.9	0.2	10.3	0.2		< 0.1	1.5
90-92.5	0.4	1.6	0.2	9.0	**4.5**	12.7			0.2	1.4	0.1	1.6
92.5-95	0.8	1.8	0.2	10.2	**0.3**	9.2			0.1		< 0.1	1.8
95-97.5	0.2	1.3	0.1	9.8	0.5	9.9			0.2	1.6	0.3	1.7
97.5-100	0.1	1.6	0.2	8.3	0.5	10.8			0.2		0.2	1.2
100-102.5	0.2	1.2	< 0.1	10.8	0.4	10.9			0.2	1.5		
102.5-105	0.3	1.2	0.2	7.9	0.4	10.4			0.2			
105-107.5	0.6	1.4	0.2	9.3	0.3	10.3			0.5	1.1		
107.5-110	0.2	1.8	0.6	7.2	0.3	8.1			0.2			
110-112.5			0.2	9.5		8.8						
112.5-115			0.2	6.0								

[a]Pt values in red for Kolb and Flamingo Bay are average values for duplicate samples shown in Table S1.
[b]For Pen Point, grain size data was collected in 5 cm intervals.

Supplementary Information Table 10. Pt concentrations for tephra/ash from five volcanoes.

Analyte Symbol	Au	Pt	Pt	Pd	Pt/Pd	MATERIAL	VOLCANO	LOCATION	COUNTRY	Lat	Long
Unit Symbol	ppb	ppb	ppb	ppb	ratio						
Detection Limit	1	0.1	0.1	0.1							
Analysis Method	FA-MS	FA-MS	FA-MS	FA-MS							
/Sample											
TEPH-SPURR	6	< 0.1	0.1	0.6	0.17	Tephra-ash	Mount Spurr	Alaska	US	~61.20	-152.1
TEPH-KAM	3	< 0.1	0.1	0.8	0.13	Tephra-ash	Shiveluch Volcano	Kamchatka Penin.	Russia	~56.50	161.3
TEPH-TOW	3	< 0.1	0.1	0.3	0.33	Pumice	Towada Caldera	Akita Prefecture	Japan	~40.45	140.9
TEPH-HONO	2	3.5	3.5	2.5	1.40	Tephra-ash	Diamond Head	Oahu, Hawaii	US	~21.25	-157.8
TEPH-LST	1	< 0.1	0.1	0.3	0.33	Tephra-ash	Laacher See	Reinhausen	Germany	~51.46	10.0
